# Relative importance of composition structures and biologically meaningful logics in bipartite Boolean models of gene regulation

**DOI:** 10.1038/s41598-022-22654-7

**Published:** 2022-10-28

**Authors:** Yasharth Yadav, Ajay Subbaroyan, Olivier C. Martin, Areejit Samal

**Affiliations:** 1 The Institute of Mathematical Sciences (IMSc), Chennai, 600113 India; 2 Homi Bhabha National Institute (HBNI), Mumbai, 400094 India; 3 Université Paris-Saclay, CNRS, INRAE, Univ Evry, Institute of Plant Sciences Paris-Saclay (IPS2), 91190 Gif sur Yvette, France; 4 Université Paris Cité, CNRS, INRAE, Institute of Plant Sciences Paris-Saclay (IPS2), 91190 Gif sur Yvette, France

**Keywords:** Complexity, Regulatory networks

## Abstract

Boolean networks have been widely used to model gene networks. However, such models are coarse-grained to an extent that they abstract away molecular specificities of gene regulation. Alternatively, *bipartite* Boolean network models of gene regulation explicitly distinguish genes from transcription factors (TFs). In such bipartite models, multiple TFs may simultaneously contribute to gene regulation by forming heteromeric complexes, thus giving rise to *composition structures*. Since bipartite Boolean models are relatively recent, an empirical investigation of their biological plausibility is lacking. Here, we estimate the prevalence of composition structures arising through heteromeric complexes. Moreover, we present an additional mechanism where composition structures may arise as a result of multiple TFs binding to *cis*-regulatory regions and provide empirical support for this mechanism. Next, we compare the restriction in BFs imposed by composition structures and by biologically meaningful properties. We find that though composition structures can severely restrict the number of Boolean functions (BFs) driving a gene, the two types of minimally complex BFs, namely nested canalyzing functions (NCFs) and read-once functions (RoFs), are comparatively more restrictive. Finally, we find that composition structures are highly enriched in real networks, but this enrichment most likely comes from NCFs and RoFs.

## Introduction

**Figure 1:**
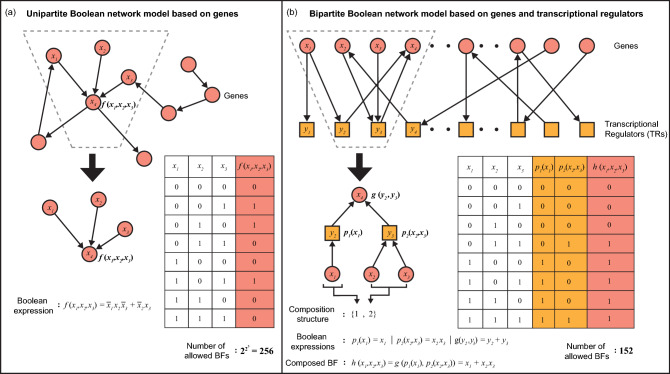
Boolean functions in unipartite versus bipartite network models of transcriptional gene regulation. (**a**) A unipartite Boolean network model consisting of only genes. The dashed trapezium highlights a subgraph wherein 3 genes with expression states $x_1, x_2\ \text {and}\ x_3$, directly regulate the gene with expression state $x_4$. Thus, the BF *f* determining the state $x_4$ of the output gene depends on the states of the 3 input genes $x_1, x_2\ \text {and}\ x_3$, and the truth table for this 3-input BF *f* is shown in the figure; its “bias”, defined as the number of 1’s in the output column, is 3 for this case. Note that any one of the $2^{2^3} = 256$ possible 3-input BFs can be assigned to BF *f*. (**b**) A bipartite Boolean network model accounting for the two types of molecular species involved in transcriptional regulation namely, the genes and transcriptional regulators (TRs). In this bipartite Boolean network model, the states of genes are denoted by variables $x_1, x_2, \ldots , x_i$ and the states of TRs are denoted by variables $y_1, y_2, \ldots , y_j$. The dashed trapezium highlights the subgraph wherein the gene with state $x_1$ determines the TR with state $y_2$ according to a 1-input BF $p_1(x_1) = x_1$, and the genes with states $x_2$ and $x_3$ determine the state of the TR $y_3$ according to a 2-input BF $p_2(x_2, x_3) = x_2x_3$. Moreover, the TRs with states $y_2$ and $y_3$ in this subgraph directly regulate the gene with state $x_4$ according to a 2-input BF $g(y_2, y_3) = y_2 + y_3$. Ultimately, the regulation of the output gene with state $x_4$ depends on the states of the input genes $x_1, x_2\ \text {and}\ x_3$ according to a 3-input BF $h(x_1, x_2, x_3) = g(p_1(x_1),p_2(x_2,x_3)) = x_1 + x_2 x_3$. Fink and Hannam^47^ called such a subgraph a “composition structure” and the BF *h* corresponding to the subgraph a “composed BF”. The truth table of a composed BF *h* allowed by this particular composition structure {1, 2} is shown in the figure. Moreover, for the composition structure $\{1, 2\}$, there are $2^{2^1}2^{2^2}2^{2^2} = 256$ ways to combine the BFs *g*, $p_1$ and $p_2$ and these combinations result in only 152 unique BFs *h* after accounting for the permutations of the inputs $x_1$, $x_2$ and $x_3$ (see “Methods”).

Transcriptional regulation is a fundamental mechanism for the control of gene expression^1^. Significant research in systems biology has thus been focused on reconstruction and analysis of transcriptional regulatory networks^2–5^. Boolean modeling is a widely used framework for studying the dynamics of such gene regulatory networks (see Fig. 1a). Stuart Kauffman^6,7^ and René Thomas^8,9^ pioneered the use of Boolean models to better understand the dynamical behaviour of gene networks including fixed points and cyclic attractors^4,10,12^. Specifically, dynamical models based on Boolean networks can provide a powerful means to explain the most important properties of cell differentiation^11^. Over time Boolean modeling has gained a wide appeal and has thus been extended to capture the dynamics of other types of biological networks such as signalling^13^ and metabolic networks^14–16^.

Until the turn of this century, the paucity of empirical data on the structure (including combinatorial regulation) of real biological networks mandated a statistical approach based on ensembles of random Boolean networks for probing the dynamics of gene regulatory networks^6,10,17–19^. Random Boolean networks are typically defined by placing interactions (directed edges) between randomly chosen genes (nodes) and assigning random logical update rules to those nodes. However, mounting evidence over the past two decades, obtained via biological network reconstruction using large-scale data from high-throughput experiments^3,20,21^, has shown that the architecture of real gene networks is far from random, both for their network structure^3,5,15,20,22–25^ and for their logical update rules^12,15,26–33^, i.e., the Boolean functions (BFs) assigned to each associated gene.

### Biologically meaningful Boolean functions

Further investigation into the nature of Boolean functions capturing gene regulation revealed that certain classes of regulatory Boolean logics are particularly biologically meaningful. These types of BFs have been classified as unate functions (UFs)^34^, canalyzing functions (CFs)^35^ and nested canalyzing functions (NCFs)^10,36,37^. Stuart Kauffman, in his book titled “The Origins of Order”^35^, propounded the idea that canalyzing functions reflect the “chemical simplicity” of the underlying molecular regulatory mechanisms in gene networks. Using Kauffman’s proposition as a premise, Subbaroyan et al.^38^ recently showed that NCFs and Read-once functions (RoFs) minimize two notions of complexity of Boolean functions, namely, the average sensitivity and the Boolean complexity, respectively. This effort has led to the addition of a new type of BF previously unexplored in Boolean models of living systems, the Read-once function (RoF), to the list of biologically meaningful BFs. Remarkably, the ‘simplest’ logics, NCFs and RoFs, also form the most restrictive subset of BFs among other biologically meaningful BFs in the space of all BFs^38^.

### Bipartite Boolean networks

Evidently, the Boolean network model of gene regulation is a coarse-grained picture of biological reality. There have been proposals to incorporate more realistic features within the Boolean framework^39–44^. Graudenzi et al.^39^ were the first to propose a bipartite Boolean network model of gene regulation with an aim to incorporate more realistic assumptions about the timescales of genetic processes. Remarkably, their model, called as gene protein Boolean network or gene product Boolean network (GPBN), could explicitly capture the interactions between genes and proteins, or genes and gene products (e.g., microRNAs), respectively. Hannam et al.^44^ generalized the notion of GPBNs further and proposed a bipartite Boolean network model that could also account for the formation of heteromeric protein complexes in regulatory processes. More precisely, the biological basis behind such a bipartite model of transcriptional regulation is as follows^44,45^. Firstly, a factor affecting a gene’s transcription rate can be either a single TF or a complex of TFs (e.g. heterodimer of TFs^46^). We refer to either type as a transcriptional regulator (TR). Thus the presence of a TR may depend on the expression of one or more genes. Secondly, multiple TRs can control the expression of a given gene. Note that genes are regulated not only by transcription factors but by other types of molecules such as miRNAs and hormones, and accounting for these in the bipartite formalism proposed by Fink and Hannam^47^ requires a further exploration of the framework. We do believe however that it may be possible to explicitly account for complexes containing different molecules such as RNA-binding proteins or hormone-receptor complexes in this framework but do not pursue this further in this work. Fink and Hannam^47^ capture the gene-TF-gene interactions in bipartite Boolean networks via subgraphs called *composition structures* and elucidate how composition structures allow for a *composition* of BFs to be defined on genes. Further, they show that the presence of composition structures can severely restrict the space of allowed BFs. Note that the restrictive nature of the composition of BFs, albeit in unipartite Boolean models, has also been considered by Shmulevich et al.^48^.

### Composition structures

Fink and Hannam^47^ introduced the term *composition structure* to denote specific subgraphs of gene-TF-gene interactions in a bipartite Boolean network. More precisely, a composition structure $\{t_1, t_2, \ldots , t_r\}$ is assigned to a given gene if its transcriptional regulation depends on the states of *r* TRs according to a BF of *r* inputs. Further, the state of each TR *i*, where $i \in [1,r]$, in turn depends on the states of $t_i$ genes according to a BF of $t_i$ inputs. See “Methods” for a formal definition of composition structures.

Figure 1b illustrates the composition structure $\{ 1,2 \}$ and its corresponding composed BF arising in a bipartite Boolean network. Here, the state of a given gene $x_4$ depends on its input TRs $y_2$ and $y_3$ according to a 2-input BF $g(y_2,y_3)$. Further, $y_2$ depends on the input gene $x_1$ according to a 1-input BF $p_1(x_1)$, and $y_3$ depends on input genes $x_2$ and $x_3$ according to a 2-input BF $p_2(x_2,x_3)$. Thus, in this composition structure $\{ 1,2 \}$, the state of $x_4$ ultimately depends on $x_1, x_2$ and $x_3$ according to a composed BF of 3-inputs $h(x_1, x_2, x_3) = g(p_1(x_1),p_2(x_2,x_3))$. Such a composition of BFs reduces the number of allowed 3-input BFs. Note that there are two other composition structures possible for 3 inputs, namely $\{1,1,1\}$ and $\{3\}$, which do not restrict the space of allowed BFs. Hence, these two composition structures can be considered as *trivial* whereas $\{ 1,2 \}$ can be considered as a *non-trivial* composition structure (see “Methods”). Finally, composition structures also allow for autoregulation, an important feature in determining the attractor landscape^49^, wherein a TF associated with a gene can regulate the expression of that same gene.

### Motivation and objectives

Currently, the bipartite Boolean models proposed^44,45,47^ for transcriptional gene regulation are theoretical propositions without a solid grounding in empirical evidence. Our work approaches the question of prevalence of composition structures in real gene regulatory networks from a data-centric perspective. The central theme of our work is thus to examine how plausible it is for both composition structures and composed BFs to occur in real transcriptional regulatory networks by analyzing published experimental data. We begin by estimating the prevalence of composition structures arising in two different scenarios of gene regulation. The first scenario is gene regulation by heteromeric protein complexes which act as transcription regulators^44,45^. The other scenario, which is a novel aspect of this work, accounts for transcriptional regulation via *cis*-regulatory elements, in particular promoters and enhancers^50,51^, that can be bound by transcription factors.

Next, we will build upon the work of Fink and Hannam^47^ on Boolean compositions and augment their approach for counting the number of possible BFs under Boolean compositions by accounting for the fact that the different input variables are distinguishable and so are non-equivalent under permutation. We then compare the restriction in the logic rules in gene regulatory networks due to Boolean compositions with the restriction due to different types of biologically meaningful BFs, and thereafter analyze how often Boolean compositions display biologically meaningful properties. Finally, we evaluate the enrichment (depletion) and *relative* enrichment (depletion) of composed BFs in a compiled empirical dataset of 2687 BFs from published reconstructed Boolean models of biological systems.

## Results

### Quantifying the presence of protein complexes that can act as transcriptional regulators

Hannam et al.^44^ proposed to model transcriptional gene regulation via bipartite Boolean networks, thus allowing them to distinguish genes from proteins or protein complexes. Non-trivial composition structures arise if regulation requires complexes of TFs^44,45^ (see “Methods”). To our knowledge there has been no systematic study of such complexes in any organism, and we here attempt to fill this gap.

Genes often come in families following either segmental or whole genome duplications, and that is the case in particular for those coding for TFs. There are several organisms where it has been shown that TFs within a given family form complexes in the form of heterodimers or even multimers contributing to gene regulation^52–55^. For instance the family of TFs called *auxin response factors* (ARFs) includes over 20 members in numerous plants and it has been shown that they form heterodimers that activate gene transcription^54,56^. However, a quantitative assessment of the frequency at which heteromeric complexes contribute to gene regulation has not been carried out. The prevalence of such complexes in real-world gene regulatory networks can provide empirical support for the (frequent or not) occurrence of non-trivial composition structures.

We obtained a list of 1325 macromolecular complexes in *H. sapiens* from the EBI Complex Portal database^57^, and the list of 1639 human TFs from http://humantfs.ccbr.utoronto.ca/ provided by Lambert et al.^58^. Among the 1639 human TFs, we selected only those TFs that were reviewed in the SWISS-PROT^59^ protein database, resulting in a list of 1617 human TFs that was used for further analysis. We found that among the 1325 complexes in *H. sapiens*, 169 satisfy the constraint of being heteromeric with all subunits corresponding to TFs (Supplementary Table S1). Of those, 165 are heterodimers and the remaining 4 are heterotrimers. Furthermore, there are 84 unique TFs composing these 169 complexes. Second, we manually searched for DNA binding evidence for each of these 169 heteromeric complexes and found that DNA binding has been verified for 86 of them. This then leaves us with 86 validated complexes of TFs that act as TRs, and thus, are likely candidates for forming composition structures.

Another approach we take to estimate the number of protein complexes acting as transcriptional regulators is to determine from the literature if there are transcription factor families known to form heterodimers. Two genes coding for a protein can derive from a common ancestor (by duplication) leading to paralogs, and in particular to proteins with similar sequences, structure and function. Thus the complex forming propensity of a TF is expected to be conserved across the elements in that particular family. In evolution, this phenomenon is so common that one often has dozens or more genes belonging to the same family. We thus explored the specific importance of heteromers of TFs belonging to particular families. Indeed, it is known that certain classes of TFs, for instance basic leucine zipper (bZIP)^53,60^ and basic helix-loop-helix (bHLH)^61^ classes, bind to DNA as homo- or hetero-dimers^55,62,63^. Knowing the prevalence of such TFs could shed light on the abundance of dimeric complexes which act as TRs. Thus for the 1617 TFs in *H. sapiens* we used the JASPAR database^64^ to obtain the associated TF families; JASPAR provides a manually curated list of DNA transcription factor binding motifs, the corresponding TFs, family information etc. Focusing on the TFs of the bZIP and bHLH families, we found 36 TFs in the first family and 38 in the second family (see Supplementary Table S2). Although our current data suggests that TF complexes are not so prevalent, we cannot rule out that this conclusion is an artifact of insufficient experimental evidence on complexes regulating genes. A similar count of complexes involved in transcriptional regulation in *Saccharomyces cerevisiae* is presented in the Supplementary Information and Supplementary Tables S3 and S4. That system leads us to a similar conclusion as the one made from complexes in humans.

### Composition structures arising through enhancers

Bipartite Boolean network models provide a quite general framework and so for instance composition structures can accommodate other mechanisms of eukaryotic gene regulation than the one involving complexes as covered in the previous sub-section. Here, we propose one such alternative picture where the intermediate transcriptional regulators (TRs) are no longer protein complexes but are associated with *cis*-regulatory modules such as promoters, enhancers, or insulators. In eukaryotes, transcription is typically regulated via the binding of TFs upstream of the gene^50,51,65^. Promoters are located close to the transcription start site where RNA polymerases and transcription factors assemble to initiate transcription^66^. Enhancers on the other hand may be located at rather large distances (in fact both upstream or downstream) of the target gene they regulate^67^. Enhancers are “active” or “inactive” based on whether their chromatin state is accessible or not; in the former case, transcription factor binding sites within these enhancers can attract specific TFs and thus modulate transcription of nearby genes^51^. Interestingly, a given enhancer typically contains multiple such binding sites and is thus considered to be a *cis*-regulatory module^51,68^.

**Figure 2:**
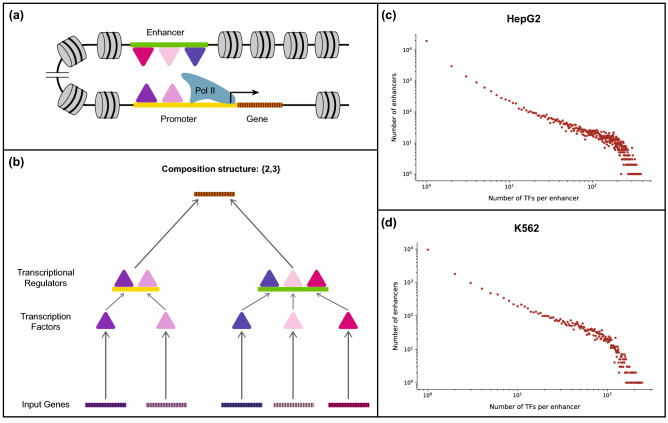
Non-trivial composition structures arising due to enhancers bound by multiple transcription factors. (**a**) A biologically plausible mechanism revealing the occurrence of non-trivial composition structures in transcriptional gene regulation. Multiple transcription factors (TFs) can bind to the promoter as well as the enhancer region(s) of a target gene. The enhancers and promoters bound by TFs then act as transcriptional regulators (TRs) of their target genes, resulting in non-trivial composition structures. (**b**) A schematic representation of the composition structure $\{ 2, 3 \}$ arising in subfigure (**a**). The target gene is regulated by an active promoter that is bound by 2 TFs, and an active enhancer that is bound by 3 TFs. (**c**) Scatter plot showing the number of active enhancers bound by a given number of TFs in the HepG2 cell line in humans. We found that 32.68% of the active enhancers in HepG2 are bound by at least 2 TFs. (**d**) Scatter plot showing the number of active enhancers bound by a given number of TFs in the K562 cell line in humans. We found that 44.31% of the active enhancers in K562 are bound by at least 2 TFs. The *x* and *y* axes in part (**c**) and (**d**) are in $\log$ scale. These results suggest that non-trivial composition structures are prevalent in gene regulatory networks.

Figure 2a and b illustrate how enhancers and promoters may act as TRs in the composition structure $\{2, 3\}$ where we have chosen to have 2 TFs binding to the promoter and 3 TFs binding to the enhancer. One can suppose that an abundance of enhancers containing multiple TF binding sites is suggestive of the prevalence of non-trivial composition structures in real-world gene regulatory networks. In view of this possibility, we perform an analysis to provide a quantitative estimate of the number of TFs that bind to active enhancers in two widely-studied human cell lines namely, HepG2 and K562.

For the cell line HepG2, we used ChIP-seq peaks provided for 458 unique TFs and a total of 32929 enhancers detected as active (see “Methods”). 2976 enhancers had exactly one TF binding within their region while 10754 enhancers had two or more TFs binding within their region, representing $32.68\%$ of the total number of enhancers in HepG2 (Fig. 2c, Supplementary Fig. S1(a)). Additionally, of the 458 TFs for which data is available in HepG2, we found that 456 TFs bind to at least one of the enhancers detected as active. For the cell line K562, we used ChIP-seq peaks provided for 323 unique TFs and a total of 20471 enhancers detected as active. 1801 enhancers had exactly one TF binding within their region while 9071 enhancers had two or more TFs binding within their region, representing $44.31\%$ of the total number of enhancers in K562 (Fig. 2d, Supplementary Fig. S1(b)). Additionally, of the 323 TFs for which data is available in K562, we found that 322 TFs bind to at least one of the enhancers detected as active. The fact that $32.68\%$ and $44.31\%$ of the active enhancers in HepG2 and K562, respectively can be bound by at least two TFs suggest that non-trivial composition structures indeed do arise frequently in gene regulatory logics.

### Accounting for all possible permutations of the inputs in a composition structure

In their procedure to count BFs arising from a composition structure, Fink and Hannam^47^ do not account for permutations of the input variables, that is they ignore the labels of the inputs. In the present work, we have extended Fink and Hannam’s counting approach by accounting for all the permutations of input variables in a given composition structure (see “Methods”). Including all possible permutations of inputs is sufficient to ensure that all isomorphisms (i.e., permutations and negations of inputs) of a BF in a composition structure are also present therein (see “Methods” for definition of isomorphism of BFs). Table 1 provides a comparison of the number of distinct BFs allowed by different composition structures for $k\le 5$ inputs, both with and without including all possible permutations of the input variables. Table 1 also provides these results as fractions among all possible BFs for $k\le 5$ inputs. Naturally, we find that accounting for all possible permutations of inputs increases the number of BFs in a composition structure in comparison to those reported by Fink and Hannam^47^. However, this does not alter the central result of Fink and Hannam^47^, that is, composition structures significantly restrict the space of possible BFs. This is evident from the trends for the fractions of composed BFs among all possible BFs as a function of the number of inputs (see Table 1).

**Table 1:** Comparison of the number and fraction of BFs allowed by different composition structures, with and without including all possible permutations of input variables.

Inputs (*k*)	Composition structure	Number of composed BFs		Fraction of composed BFs	
		Without permutation	With permutation	Without permutation	With permutation
1	{1}	4	4	1	1
2	{2}	16	16	1	1
	{1,1}	16	16	1	1
3	{3}	256	256	1	1
	{1,2}	88	152	0.344	0.594
	{1,1,1}	256	256	1	1
4	{4}	65,536	65,536	1	1
	{1,3}	1528	4864	0.023	0.074
	{2,2}	520	1208	0.008	0.018
	{1,1,2}	1696	6216	0.026	0.095
	{1,1,1,1}	65,536	65,536	1	1
5	{5}	4,294,967,296	4,294,967,296	1	1
	{1,4}	393,208	1,921,928	$9.16 \times 10^{-05}$	$4.47 \times 10^{-04}$
	{2,3}	9160	71,608	$2.13 \times 10^{-06}$	$1.67 \times 10^{-05}$
	{1,1,3}	30,496	263,488	$7.10 \times 10^{-06}$	$6.13 \times 10^{-05}$
	{1,2,2}	11,344	100,768	$2.64 \times 10^{-06}$	$2.35 \times 10^{-05}$
	{1,1,1,2}	457,216	3,446,488	$1.06 \times 10^{-04}$	$8.02 \times 10^{-04}$
	{1,1,1,1,1}	4,294,967,296	4,294,967,296	1	1

The composition structures in the bipartite Boolean network framework of transcriptional gene regulation are categorized based on the number of inputs *k* to a gene in the corresponding unipartite Boolean network framework. The column “Number of composed BFs” gives the number of distinct BFs in a composition structure, and the subcolumns provide a comparison of the number of such BFs both without and with the accounting for all possible permutations of the input variables. The column “Fraction of composed BFs” gives the fraction of distinct BFs in a composition structure among all possible BFs for a given number *k* of inputs, and the subcolumns provide a comparison of the fraction of such BFs both without and with the accounting for all possible permutations of the input variables.

### Overlap of Boolean functions across various *k*-input composition structures

There are multiple composition structures $\{t_1,\ldots , t_r\}$ possible for a given number of inputs *k* such that $t_1 + t_2 + \cdots + t_r = k$, and each composition structure allows a certain set of BFs. However, composed BFs can belong to more than one composition structure. Therefore, it is worthwhile to examine the overlaps of composed BFs across all non-trivial composition structures with a given number of inputs *k*. Here, we analysed the intersections of composed BFs across non-trivial composition structures for $k=4$ and $k=5$ inputs. We reiterate that there are no non-trivial composition structures for $k=1$ and $k=2$ inputs, and note that {1, 2} is the only non-trivial composition structure for $k=3$.

**Figure 3:**
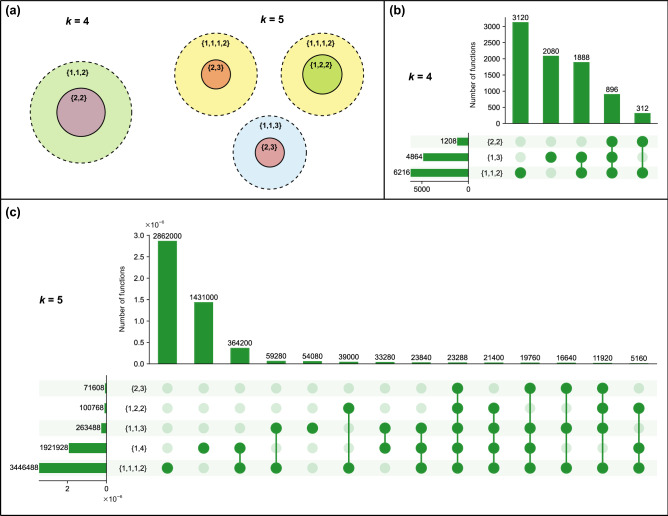
Overlaps between the sets of BFs compatible with different composition structures at $k=4$ and $k=5$ inputs. (**a**) Venn diagrams illustrating *proper* subsets among the sets of non-trivial composition structures at $k=4$ and $k=5$ inputs. (**b**) UpSet plot illustrating the number of BFs that are present in all possible intersections of non-trivial composition structures at $k=4$ inputs. (**c**) UpSet plot illustrating the number of BFs that are present in all possible intersections of non-trivial composition structures at $k=5$ inputs. The horizontal bars in the UpSet plots indicate the number of BFs that are present in different composition structures. The vertical bars indicate the number of BFs that are simultaneously present in some and absent from other composition structures, as specified by the underlying dark and light green circles.

For $k=4$ inputs, we find that the set of BFs in the composition structure $\{ 2, 2\}$ is a proper subset of the set of BFs in $\{ 1, 1, 2\}$ (see Fig. 3a). For $k=5$ inputs, we find that the set of BFs in the composition structure $\{ 2, 3\}$ is a proper subset of the set of BFs in $\{ 1, 1, 3\}$ as well as $\{ 1, 1, 1, 2\}$, and the set of BFs in the composition structure $\{ 1, 2, 2\}$ is a proper subset of the set of BFs in $\{ 1, 1, 1, 2\}$ (see Fig. 3a). Further, we give the number of BFs in all possible intersections of non-trivial composition structures for $k=4$ and $k=5$ inputs through UpSet plots^69^ in Fig. 3b and c, respectively.

### Comparing restriction levels: composition structures versus biologically meaningful types

Clearly, imposing a non-trivial composition structure significantly restricts the space of allowed BFs within the complete space of BFs with *k* inputs. As shown by some of us recently^38^, the same holds when imposing certain biologically meaningful properties. Here, we compare the level of restriction achieved by four established biologically meaningful types of BFs, namely unate functions (UFs), canalyzing functions (CFs), nested canalyzing functions (NCFs) and read-once functions (RoFs), to that achieved by composed BFs of a given composition structure, in the space of all BFs with *k* inputs. See “Methods” for the formal definitions of biologically meaningful BFs. Among the four different types of biologically meaningful BFs, it is known that the NCFs represent the smallest fraction in the space of all BFs^38^. For $k=4$ and $k=5$ inputs, we find that certain composition structures restrict very strongly, though less than NCFs (see Supplementary Table S5). Specifically, at $k=4$ inputs, $\{ 2, 2 \}$ is the most restrictive one. The composed BFs in $\{ 2, 2 \}$ occupy a fraction of 0.018 among all BFs, which is 1.63 times greater than the fraction occupied by NCFs at $k=4$ (whose value is 0.011). For $k=5$ inputs, $\{ 2, 3 \}$ is the most restrictive composition structure. The BFs in $\{ 2, 3 \}$ occupy a fraction of $1.67 \times 10^{-5}$, which is about 6.76 times greater than the fraction occupied by NCFs at $k=5$ (whose value is $2.47 \times 10^{-6}$). In Supplementary Table S5, we compare the fraction of BFs in the most restrictive composition structure to the fractions for each of the four types of biologically meaningful BFs for $k \le 5$ inputs.

We next evaluated how often a BF in a composition structure also displays biologically meaningful properties. Table 2 shows the number of composed BFs that belong to each of the four types of biologically meaningful BFs for non-trivial composition structures with $k \le 5$ inputs. Clearly imposing BFs to be biologically meaningful and to be compatible with a given composition structure severely restricts the possible BFs. We also find that certain types of biologically meaningful BFs, in particular NCFs, are proper subsets of BFs in certain composition structures. Specifically, all the 64 NCFs with $k=3$ inputs are contained in the composition structure $\{1,2\}$, all the 736 NCFs with $k=4$ inputs are contained in the composition structures $\{1,3\}$ and $\{1,1,2\}$, and all the 10624 NCFs with $k=5$ inputs are contained in the composition structures $\{1,4\}$, $\{1,1,3\}$ and $\{1,1,1,2\}$. Moreover, all CFs with $k = 3,\ 4,\ \text {and}\ 5$ inputs are a subset of the composition structures $\{1,2\}$, $\{1,3\}$ and $\{1,4\}$, respectively, whereas all RoFs with $k = 4$ and 5 inputs are a subset of the composition structures $\{1,1,2\}$ and $\{1,1,1,2\}$, respectively. In Supplementary Table S6, we provide the fraction of composed BFs that belong to each of the four types of biologically meaningful BFs for non-trivial composition structures with $k\le 5$ inputs.

**Table 2:** Number of BFs in different composition structures that display biologically meaningful properties.

Composition structure	Number of composed BFs	Number of biologically meaningful BFs in composition structure			
		UF	CF	NCF	RoF
{1,2}	152	96	120	64	64
{1,3}	4864	1210	3514	736	736
{2,2}	1208	634	730	224	320
{1,1,2}	6216	1370	1850	736	832
{1,4}	1,921,928	41,676	1,292,276	10,624	12,544
{2,3}	71,608	13,676	33,596	3264	6784
{1,1,3}	263,488	26,156	80,996	10,624	14,144
{1,2,2}	100,768	17,836	25,236	5504	9984
{1,1,1,2}	3,446,488	61,516	122,516	10,624	15,104

The number of BFs within a non-trivial composition structure that also belong to each of the four types of biologically meaningful functions, namely Unate functions (UFs), Canalyzing functions (CFs), Nested canalyzing functions (NCFs) and Read-once functions (RoFs). The column “Number of composed BFs” gives the number of BFs that are allowed in a given composition structure.

We also computed the number and fraction of composed BFs for different composition structures which have odd bias. Recently, some of us showed that BFs with odd bias are preponderant among BFs in reconstructed Boolean network models of biological systems^38^. Furthermore, it was shown that NCFs^70^ and RoFs^38^ have odd bias. Here, we find that the fraction of BFs with odd bias in any composition structure with $k\le 5$ inputs is less than 0.5 (see Supplementary Table S7). Additionally, we find that BFs – with any given even bias – occur in all composition structures with $k\le 5$ inputs. In Supplementary Table S7, we list the odd biases of BFs that are present in composition structures with $k\le 5$ inputs.

### Enrichments of composed BFs in reconstructed biological networks

In this section, we present the results of our analyses of the abundances of composed BFs in a compiled reference biological dataset of 2687 BFs from 88 published Boolean network models of biological systems^38^. More explicitly, we do not reconstruct the composition structures associated with each of the 2687 BFs in our database, but rather determine which composition structure each of the 2687 BFs belong to, by comparing the real BFs with the composed BFs of various composition structures. In the “Methods” section, we provide details about the compilation and curation of this empirical dataset.

**Figure 4:**
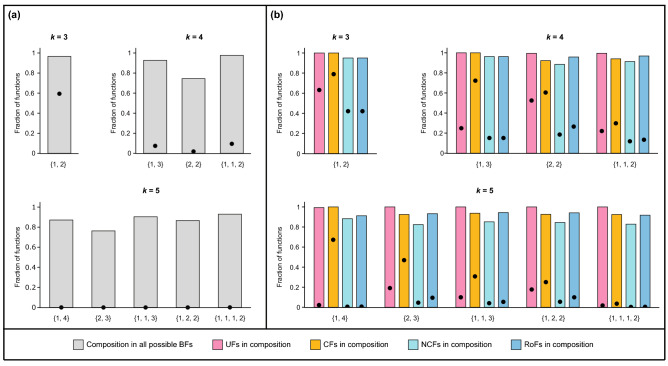
Abundance of composed BFs in reconstructed biological networks. (**a**) Bar plots give the fraction of BFs in the reference biological dataset that are compatible with each of the composition structures. The black dots indicate the fraction when considering all possible BFs instead of only the ones in the reference biological dataset. Note that since the sets of BFs allowed by different composition structures overlap with each other, the sum of the bar plot values may be larger than 1. (**b**) For all BFs of the reference biological dataset compatible with a given composition structure, the bars give the fraction of these BFs that belong to each of the four biologically meaningful sub-types: Unate functions (UFs), Canalyzing functions (CFs), Nested canalyzing functions (NCFs), and Read-once functions (RoFs). Again, the black dots give these fractions when considering instead all possible BFs.

To begin, we computed two proportions for each possible composition structure. The first is the proportion of BFs with *k* inputs in the reconstructed biological networks that belong to the given composition structure (bar plots in Fig. 4a). The second is the corresponding proportion in the “random ensemble” with *k* inputs; that proportion is thus given by the number of BFs with *k* inputs that are compatible with the given composition structure, divided by the total number of BFs (black dots in Fig. 4a). The results show that the proportions of composed BFs in the *reference biological dataset* are larger than in the ensemble of random BFs, for each composition structure, indicating that non-trivial composed BFs are enriched in real biological networks. Note that the sets of BFs allowed by different composition structures overlap with each other (see Fig. 3), allowing for the sum of the height of the bars in Fig. 4a to be larger than 1. To consider this question in greater depth, we define the “enrichment factor” as the ratio of the first and the second proportions. For instance, for the composition structures $\{ 2, 2 \}$ and $\{ 2, 3 \}$ that are the most restrictive composition structures for $k=4$ and $k=5$ inputs, the corresponding enrichment factors are 40.37 and 45760.08. To check the level of significance of this effect, we applied a standard statistical test (see “Methods”). In Supplementary Table S8, we list the enrichment factors for all non-trivial composition structures having $k \le 5$ inputs and we give the corresponding one-sided *p* values. These *p* values show that the enrichment effects are indeed statistically significant, providing evidence in biological systems of a selection pressure in favor of each of the non-trivial composition structures.

Figure 4b is a bar plot of the fractions in the reference biological dataset of the four biologically meaningful sub-types when focusing on the BFs satisfying a given composition structure. In addition, the black dots give the corresponding fractions when using the random ensemble instead of the reference biological dataset. We call “relative enrichment” $E_R$ the ratio of these fractions that focuses on both a given composition structure and a given biologically meaningful sub-type of BF. The $E_R$s are larger than 1 for all non-trivial composition structures with number of inputs $k \le 5$, suggesting that the four biologically meaningful sub-types of composed BFs are enriched within any composition structure in the reference biological dataset. Table 3 gives the $E_R$ values for the four biologically meaningful sub-types in all non-trivial composition structures with number of inputs $k \le 5$. Furthermore, the computed relative enrichment values are statistically significant as determined by one-sided *p* values (see Supplementary Table S9).

**Table 3:** Relative enrichment of biologically meaningful BFs among composed BFs of different composition structures in the reference biological dataset. This table gives the relative enrichment values $E_R$ in the reference biological dataset for the four biologically meaningful sub-types within composed BFs for different non-trivial composition structures with number of inputs $k \le 5$. These four biologically meaningful sub-types within composed BFs include those BFs in a composition structure that also happen to be Unate functions (UFs), Canalyzing functions (CFs), Nested canalyzing functions (NCFs), or Read-once functions (RoFs).

Composition structure	$E_R$ of biologically meaningful sub-types in a given composition structure			
	UF	CF	NCF	RoF
{1,2}	1.58	1.27	2.26	2.26
{1,3}	4.02	1.38	6.36	6.36
{2,2}	1.90	1.53	4.77	3.62
{1,1,2}	4.52	3.16	7.71	7.23
{1,4}	45.78	1.49	159.62	139.70
{2,3}	5.24	1.97	18.07	9.85
{1,1,3}	10.07	3.05	21.11	17.57
{1,2,2}	5.65	3.70	15.46	9.49
{1,1,1,2}	56.03	26.00	268.47	209.30

A previous analysis^38^ showed that biologically meaningful BFs are enriched in our reference biological dataset. Notably, those enrichments are likely driven by complexity minimization, with NCFs and RoFs respectively minimizing two complexity measures namely, average sensitivity and Boolean complexity^38^. An immediate question that then arises is whether the enrichments of composed BFs as found in Fig. 4 might just be driven by enrichments of NCFs and RoFs. To examine that possibility, let $T_C$ denote the set of BFs allowed by a composition structure *C* at a given number of inputs *k*, and let $T_{NCF}$ denote the set of NCFs with *k* inputs. We have determined the enrichment factors of three disjoint sets of BFs: composed BFs that are also NCFs (i.e., $T_C \cap T_{NCF}$), composed BFs that are not NCFs (i.e., $T_C \setminus T_{NCF}$), and NCFs that are not composed BFs (i.e., $T_{NCF} \setminus T_C$). Table 4 shows the enrichment factors for these three disjoint sets of BFs, for all non-trivial composition structures with $k \le 5$ inputs. We find that the BFs belonging to the set $T_C \cap T_{NCF}$ display a very high enrichment factor. Moreover, for composition structures $\{2,2\}$, $\{2,3\}$ and $\{1,2,2\}$, we find that both the sets $T_C \setminus T_{NCF}$ and $T_{NCF} \setminus T_C$ are enriched in the biological datasets. However, the enrichment factor is much larger for the set $T_{NCF} \setminus T_C$. Finally, for the composition structures $\{1,2\}$, $\{1,3\}$, $\{1,1,2\}$, $\{1,4\}$, $\{1,1,3\}$ and $\{1,1,1,2\}$ that are a superset of the corresponding NCFs, we find that the set $T_C \setminus T_{NCF}$ is either depleted or shows a lower enrichment factor compared to the set $T_C \cap T_{NCF}$. After repeating the above analysis for RoFs to estimate the enrichment factors for $T_C \cap T_{RoF}$, $T_C \setminus T_{RoF}$ and $T_{RoF} \setminus T_C$, we find that the results are similar to those for NCFs (Table 4). Furthermore, all these enrichment factors are statistically significant as determined by one-sided *p* values (Supplementary Table S10). These results suggest that although composed BFs are subject to positive selection in real biological networks, the primary driving force for enrichment is the property of being an NCF or an RoF.

**Table 4:** Comparison between the enrichments of composed BFs and biologically meaningful BFs of minimum complexity in the reference biological dataset. The table provides the enrichment factors when composed BFs in non-trivial composition structures with $k \le 5$ inputs are compared with two classes of biologically meaningful BFs of minimum complexity namely, nested canalyzing functions (NCFs) and Read-once functions (RoFs). $T_C$ denotes the set of composed BFs allowed by a composition structure at a given number of inputs *k*, $T_{NCF}$ denotes the set of all *k*-input NCFs, and $T_{RoF}$ denotes the set of all *k*-input RoFs. $\cap$ represents the intersection of two sets and $\setminus$ represents the set-theoretic difference. “–” in the columns $T_{NCF} \setminus T_C$ or $T_{RoF} \setminus T_C$ indicates that the NCFs or RoFs are a subset of the set of BFs allowed by the composition structure.

Composition structure	$T_C \cap T_{NCF}$	$T_C \setminus T_{NCF}$	$T_{NCF} \setminus T_C$	$T_C \cap T_{RoF}$	$T_C \setminus T_{RoF}$	$T_{RoF} \setminus T_C$
{1,2}	3.67	0.14	–	3.67	0.14	–
{1,3}	79.38	0.55	–	79.38	0.55	37.04
{2,2}	192.78	5.68	29.77	146.06	2.29	29.77
{1,1,2}	79.38	1.02	–	74.49	0.38	–
{1,4}	310,977.13	230.48	–	272,157.87	173.03	96,791.63
{2,3}	826,630.05	8459.68	82,296.27	450,476.77	3397.73	72,800.54
{1,1,3}	310,977.13	2286.48	–	258,889.63	883.34	0.00
{1,2,2}	570,245.27	6069.12	32263.88	350,214.73	2426.14	32,263.88
{1,1,1,2}	310,977.13	200.33	–	242,434.78	96.28	–

We have also examined these questions for the other sub-types of biologically meaningful BFs. Supplementary Table S11 lists the corresponding enrichment factors while Supplementary Table S12 lists the associated *p* values. First, we find that the set of BFs that are UFs but not composed BFs (i.e., $T_{UF} \setminus T_C$) are enriched whereas those BFs that are composed BFs but not UFs (i.e., $T_{C} \setminus T_{UF}$) are highly depleted. This suggests that UFs could also be a possible driving factor for the enrichment of composed BFs in biological networks. Second, BFs that are composed BFs but not CFs (i.e., $T_{C} \setminus T_{CF}$) are highly enriched compared to BFs that are CFs but not composed BFs (i.e., $T_{CF} \setminus T_{C}$). Though this result provides evidence for composition structures as a driving factor for the enrichment of CFs in real biological networks, we reiterate our earlier result that this enrichment is primarily driven by the property of being NCFs or RoFs.

## Discussion

We began our empirical study into the potential biological relevance of non-trivial composition structures arising in bipartite gene regulatory networks by investigating two different scenarios. In the first we estimated the degree of occurrence of heteromeric complexes formed by DNA-binding proteins while in the second we characterized co-occurrences of TF binding sites in enhancers. Recall that a composition is called non-trivial when the number of terms in the composition structure is greater than 1 and at least one of its entries is not equal to 1 (see “Methods”).

TF complexes have previously been studied in unipartite Boolean models^27,71,72^. One approach to handle TF complexes in unipartite Boolean models is to create a functional topology of the original network^27^. Creation of the functional topology involves the creation of pseudonodes called complementary nodes and composite nodes. Complementary nodes are nodes which are assigned negated literals that allow for the replacement of inhibitory interactions with activatory ones. Composite nodes on the other hand represent the conjunction of their input variables (which could be actual nodes or complementary nodes). Thus composite nodes capture the TF complexes in the unipartite Boolean network. Furthermore, we note that the effective graph formalism could also be useful in dealing with protein complexes^73^. In the scenario of transcriptional regulation by heteromeric complexes as proposed by Hannam et al.^44^, a non-trivial composition structure arises when a gene is regulated by at least two transcriptional regulators, of which at least one is a heteromeric protein complex made up of at least two monomers. Composition structures can therefore be important if a substantial fraction of genes are transcriptionally regulated by protein complexes. Generally that will require many different such complexes, but one cannot exclude that just a few complexes are involved in the control of many genes. From the data on macromolecular complexes in humans obtained from the EBI Complex Portal^57^, we find that for approximately $6.5\%$ of the complexes (86 out of 1325), all of their monomeric subunits are identified as TFs. (For such an identification, we imposed that they be present in the database of 1617 human TFs from Lambert et al.^58^ and come with strong evidence for DNA binding as ascertained by manual curation of the literature.) Furthermore, we find that $4.57\%$ of the human TFs belong to the bZIP and bHLH classes that are known to bind to DNA as homodimers or heterodimers.

It is likely that the collection of complexes in the EBI Complex Portal are biased towards complexes which do not act as TRs given that the detection and characterization of heteromeric protein complexes which act as TRs is experimentally challenging. Though our empirical analysis provides some support for Hannam’s picture of heteromeric protein complexes acting as TRs, the existing data on such complexes is insufficient to quantitatively estimate the prevalence of composition structures in real-world gene regulatory networks. Another point that requires critical assessment in this picture of gene regulation is the number of logic rules that govern the formation of the heteromeric complexes. Since a heteromeric complex is a conjunction of all its monomeric subunits, the only Boolean logic rule which captures the formation of a complex is the one linking all the components by the “AND” operator. In a general bipartite Boolean network, the upper limit for the number of logics possible for the composition structure $\{t_1, t_2, \ldots , t_r\}$ is $2^{{2}^{t_1}} 2^{{2}^{t_2}}\ \ldots \ 2^{{2}^{t_r}} 2^{{2}^{r}}$, whereas if one imposes the “AND” logic for the formation of protein complexes only $2^{{2}^{r}}$ logics are possible.

The flexibility of the bipartite formalism allows us to capture a more nuanced scenario in gene regulation that involves *cis*-regulatory elements (such as enhancers and promoters) and the transcription factors (TFs) which bind to them. In our picture, a target gene is regulated by *cis*-regulatory elements which act as transcriptional regulators (TRs) and each *cis*-regulatory element acts in a way that depends on the TFs that bind therein (see Fig. 2a). Thus, a non-trivial composition structure is realized when a gene is regulated by at least two *cis*-regulatory elements, one of which is regulated by at least two TFs. We thus inferred whether non-trivial composition structures of this kind arise in gene regulatory networks by determining how often the enhancers of a gene are bound by at least two TFs. By analyzing ChIP-seq and enhancer datasets in the two human cell lines HepG2 and K562, we find that $32.68\%$ and $44.31\%$ of their respective active enhancers bind to at least two TFs. Our result suggests that composition structures with *cis*-regulatory elements acting as transcriptional regulators are likely to be prevalent in bipartite gene regulatory networks. We remark that experimental limitations do not allow for the detection of all the enhancers for a given target gene in a given cell type, preventing the identification of exact composition structures from empirical data.

Fink and Hannam^47^ showed that composition structures can severely restrict the number of Boolean logics in the space of all BFs. In the present contribution, we address many questions from the perspective of providing a comprehensive comparison between the BFs of a composition structure and the BFs that are biologically meaningful. The main questions we address are as follows: (i) How restrictive are composition structures compared to biologically meaningful logic rules? (ii) For a given number of inputs, do the BFs belonging to two different composition structures overlap? (iii) Do BFs in a given composition structure overlap with those having a biologically meaningful logic? (iv) Are composed BFs enriched in an empirical dataset of biological logic rules? First, we provide the corrected values for the number of BFs belonging to a given composition structure by accounting for all the isomorphisms for each of the composed BFs (Fink and Hannam^47^ leave these out of their work). Note that the procedure used to count the corrected values of the number of composed BFs in the present work is purely computational and is based on enumeration. Such a computational approach limits our ability to count the number of BFs belonging to most composition structures beyond $k = 5$ inputs. We then compare the degree of restrictiveness imposed by composition structures vis-a-vis biologically meaningful logic rules and find that for all inputs up to 5-input BFs, the NCFs and RoFs are more restrictive than the most restrictive composition structures. Next, we quantify the overlaps between different composition structures and find that BFs belonging to different composition structures may partially overlap but some composition structures may in fact be subsets of other composition structures, e.g. {2,2} is a subset of {1,1,2}. Following this, we quantify the overlaps between composition structures and biologically meaningful BFs. Interestingly, we find that of the 9 composition structures (up to 5-input BFs), the NCFs are a subset of 6 composition structures. The overlaps across different composition structures and the overlaps of composition structures with biologically meaningful BFs provide essential information for analysing their enrichments in real biological networks. In particular, quantifying these overlaps help us identify specific classes of composed BFs or biologically meaningful BFs that drive the enrichments of the other classes.

Our final set of analyses are a set of 3 statistical tests to identify whether composed BFs are enriched within biological logics and if so, what are the factors that drive their enrichment. First, we find that the composed BFs are indeed enriched in our reference biological dataset in comparison to the space of all BFs. Then by computing the relative enrichment of a biologically meaningful sub-type in non-trivial composition structures (for instance, the relative enrichment of NCFs when considering BFs compatible with the composition structure {2,2}), we find that these sub-types are enriched, though the cause of its enrichment could be attributed either to the property of being biologically meaningful or to the property of belonging to the composition structure. To decide between these two possibilities, we compare the relative enrichments of biologically meaningful BFs which do not belong to the composed BFs to the relative enrichment of the composed BFs which do not belong to the biologically meaningful BFs. In a nutshell, these tests confirm that the property of being minimally complex in terms of the Boolean complexity or the average sensitivity, i.e., being either an RoF or a NCF, is most likely what drives the enrichment of composition structures.

## Methods

### Boolean models of gene regulatory networks

A Boolean model of a gene regulatory network consists of nodes (or vertices) and directed links (or edges) wherein the nodes correspond to genes and a directed link towards any gene captures the regulation of that output gene by a input gene from which the link arises^6–8,35^. In a Boolean network model, the allowed states for any node are analogous to that of a switch which can be either ‘on’ or ‘off’, and therefore, the state of a node is given by a Boolean variable *x* that can take the values 1 or 0. The dynamics of any Boolean network model is determined by two factors, namely: *update rules* or *Boolean functions* (BFs) which are assigned to each node, and the update scheme employed (e.g., *synchronous*^35^ or *asynchronous*^74^). The state of a node *j* in the network with *k* inputs at time $t+1$ is given by a BF $f = f_j(x_1,x_2,\ldots ,x_{k})$ having *k* input variables, where $x_i\in \{0,1\}$ are the states of each of the *k* inputs at time *t*. This BF maps the $2^k$ different possibilities for the *k* input variables to output values 0 or 1, i.e., $f:\{0,1\}^k\mapsto \{0,1\}$.

### Useful representations and properties of Boolean functions

A BF *f* of *k* inputs can be represented *via* an algebraic expression consisting of the *k* input variables combined using the logical operators AND (e.g. $x_1 \cdot x_2$), OR (e.g. $x_1 + x_2$) and NOT (e.g. ${\overline{x}}_1$). Alternatively, *f* can be represented as a truth table with $2^k$ rows, wherein each row corresponds to a possible choice of the set of *k* input variables (see Fig. 1). The last entry of each row in the truth table gives the output value for the corresponding realisation of the input variables. Thus, a BF can also be expressed as a binary vector of size $2^k$, where each element of the vector corresponds to the output value of the corresponding row of the truth table. Some properties associated with BFs such as bias or parity, complementarity or being isomorphic^75^, may have utility in the biological context^38^. The bias of a BF *f* is the number of ones which occur in its output vector. A BF with an odd (even) bias is said to possess an odd (even) parity. The complement ${\overline{f}}$ of a BF *f* is obtained by inverting each element in its output vector^75^. For instance, if BF $f = [0,0,1,0]$, then its complement ${\overline{f}} = [1,1,0,1]$. The isomorphisms^75^ of a BF *f* are the BFs obtained by permuting and possibly negating the inputs of *f*. For instance, the 4 isomorphisms of the BF with expression $a+b$ are $a+b$ itself, $a+{\overline{b}}$, ${\overline{a}}+b$, and ${\overline{a}}+{\overline{b}}$.

### Biologically meaningful types of Boolean functions

#### Unate function (UF)

If a BF is monotonically increasing or decreasing for each input *i*, it is said to be a unate function (UF)^34^. Formally, if a BF is monotonically increasing in $x_i$, then:

1 $$\begin{aligned} \forall \ {\mathbf {x}} \in \{0, 1\}^{k}\ \text {with}\ x_{i} = 0, \, f({\mathbf {x}} + {\mathbf {e}}_{i}) \ge f({\mathbf {x}}), \end{aligned}$$

or, if it is monotonically decreasing, then:

2 $$\begin{aligned} \forall \ {\mathbf {x}} \in \{0, 1\}^{k}\ \text {with}\ x_{i} = 0, \, f({\mathbf {x}} + {\mathbf {e}}_{i}) \le f({\mathbf {x}}). \end{aligned}$$

Here ${\mathbf {e}}_{i} \in \{0, 1\}^{k}$ denotes the unit vector having entry 1 for input *i* and 0 for all others.

#### Canalyzing function (CF)

If in a BF there exists at least one input *i* (or input variable $x_{i}$) which, when fixed to 0 or 1, fixes the output value, then that BF is said to be a canalyzing function (CF)^35^. Mathematically,

3 $$\begin{aligned} f(x_{1}, x_{2}, \ldots ,x_{i-1},x_i=a,x_{i+1},\ldots ,x_{k})=b, \, \end{aligned}$$

independent of $x_j$ for $j \ne i$. Here, $x_i$ is the canalyzing input variable, *a* is the canalyzing input value, and *b* is the canalyzed output value.

#### Nested canalyzing function (NCF)

A *k*-input BF is said to be a nested canalyzing function (NCF)^10,76^ with respect to some permutation $\sigma$ on its inputs if:

4 $$\begin{aligned} f({\mathbf {x}}) = {\left\{ \begin{array}{ll} b_{1} \quad \text {if}\ x_{\sigma (1)} = a_{1},\\ b_{2} \quad \text {if}\ x_{\sigma (1)} \ne a_{1},x_{\sigma (2)} = a_{2},\\ b_{3} \quad \text {if}\ x_{\sigma (1)} \ne a_{1},x_{\sigma (2)} \ne a_{2},x_{\sigma (3)}= a_{3},\\ \vdots \\ b_{k} \quad \text {if}\ x_{\sigma (1)} \ne a_{1},x_{\sigma (2)} \ne a_{2},\ldots ,x_{\sigma (k)} = a_{k},\\ {\overline{b}}_{k} \quad \text {if}\ x_{\sigma (1)} \ne a_{1}, x_{\sigma (2)} \ne a_{2},\ldots , x_{\sigma (k)} = {\overline{a}}_{k}.\\ \end{array}\right. } \end{aligned}$$

Here, $a_{1},a_{2},\ldots ,a_{k}$ are the canalyzing input values and $b_{1},b_{2},\ldots ,b_{k}$ are the canalyzed output values. ${\overline{a}}_{k}$ and ${\overline{b}}_{k}$ denote the complements of the Boolean values $a_k$ and $b_k$, respectively.

#### Read-once function (RoF)

If a *k*-input BF *f* can be expressed only using the operators AND, OR and NOT in such a manner that each variable appears exactly once in the Boolean expression, then the BF is said to be a Read-once function (RoF)^38,77^. Mathematically, for *f* there exists a permutation $\sigma$ on $\{1,2,\ldots ,k\}$ such that after stripping all the parentheses in the Boolean expression for $f({\mathbf {x}})$, we are left with an expression of the form:

5 $$\begin{aligned} f({\mathbf {x}}) = X_{\sigma (1)} \odot X_{\sigma (2)} \odot X_{\sigma (3)} \ldots \odot X_{\sigma (k)} . \end{aligned}$$

Here, $X_{\sigma (i)} \in \{ {x_{\sigma (i)}}$, ${\overline{x}}_{\sigma (i)}\}$ and $\odot \in \{\wedge$ (AND), $\vee$ (OR)}.

### Composition structures

We provide here a formal definition of composition structures. Consider a subgraph in the bipartite network model of transcriptional regulation wherein a given gene has *r* incoming links from *r* TRs, that is, the expression of the given gene is directly controlled by *r* TRs and each of these *r* TRs in turn have $t_i$ incoming links from $t_i$ genes where $i \in [1,r]$, that is, each TR *i* is directly dependent on $t_i$ genes. In recent work, Fink and Hannam^47^ termed such a subgraph in the bipartite model as a ‘composition structure’, and denoted it as $\{t_1, t_2, \ldots , t_r\}$ (see Fig. 1b); since the composition graph is a tree of depth 2, the ordering of the degrees (i.e., $t_i$s) is arbitrary and so one can force the sequence $\{t_1, t_2, \ldots , t_r\}$ to be increasing. In their work, Fink and Hannam^47^ assumed that the $t_1, t_2, \ldots , t_r$ genes directly controlling the *r* TRs in the subgraph are distinct. Evidently, the sum $k = t_1 + t_2 + \ldots + t_r$ gives the number of genes whose products directly regulate the targeted gene in the bipartite model. In other words, this sum *k* in the bipartite model gives the number of inputs *k* to a gene in the corresponding unipartite model.

Clearly, for a given value of *k*, there are multiple composition structures possible. For instance, the possible composition structures for $k=4$ are: $\{1,1,1,1\}, \{1,1,2\}, \{1,3\}, \{2,2\}$ and $\{4\}$. Fink and Hannam^47^ refer to the subset of functions within all $2^{2^k}$ BFs resulting from the restrictions imposed by the composition structure as “composed Boolean functions”.

### Composed Boolean functions

Consider a composition structure $\{t_1, t_2, \ldots , t_r\}$ in the bipartite Boolean network framework. Let there be a gene whose transcriptional regulation depends on the states of *r* TRs according to a BF *g* with *r* inputs. We denote the BF *g* as $g = g(y_1, y_2, \ldots , y_r)$, where $y_1, y_2, \ldots , y_r$ are the states of the *r* TRs. The state of each TR *i*, where $i \in [1,r]$, in turn depends on the states of $t_i$ genes according to a BF $p_i$ with $t_i$ inputs.

Let us denote the states of the $k = t_1 + t_2 + \ldots + t_r$ genes directly controlling the *r* TRs as $x_1, \ldots , x_{t_1}, \ldots , x_{t_1+t_2}, \ldots , x_{k}$. It follows that:

$$\begin{aligned} &y_1 = p_1(x_1, \ldots , x_{t_1}), \\ &y_2 = p_2(x_{t_1 + 1}, \ldots , x_{t_1 + t_2}), \\ &\vdots \\ &y_r = p_r(x_{t_1 + t_2 + \ldots + t_{r-1} + 1}, \ldots , x_{k}). \end{aligned}$$

The regulation of a gene in the composition structure $\{t_1, t_2, \ldots , t_r\}$ ultimately depends on the states of *k* genes according to some BF *h* of *k* inputs. This BF *h* is in fact the composition of the BFs $p_1, p_2, \ldots , p_r$ fed into *g*, that is:

$$\begin{aligned} &g(y_1, y_2, \ldots , y_r) \\ &= g( p_1(x_1, \ldots , x_{t_1}), \ldots , p_r(x_{ t_1 + t_2 + t_{r-1} + 1 }, \ldots , x_k) ) \\ &= h(x_1, x_2, \ldots , x_k). \end{aligned}$$

In the above equation, the BF *h* is said to be a composed BF. There are no restrictions on the BFs that can be assigned to $p_1, p_2, \ldots , p_r$ or *g*. Therefore, the upper limit on the possible number of composed BFs *h* is:

$$\begin{aligned} 2^{{2}^{t_1}} 2^{{2}^{t_2}}\ \ldots \ 2^{{2}^{t_r}} 2^{{2}^{r}}. \end{aligned}$$

However, the $2^{{2}^{t_1}} 2^{{2}^{t_2}}\ \ldots \ 2^{{2}^{t_r}} 2^{{2}^{r}}$ BFs thereby composed are generally not all distinct, and it is necessary to remove the redundancies to obtain the set of (non-redundant) composed BFs. Such a non-redundant set of composed BFs is referred to as “biological logics” by Fink and Hannam^47^, and in the present work we will refer to this non-redundant set of BFs as simply the “composed BFs”.

From the definition of composed BFs, it follows that if a BF *h* is associated with a composition structure $\{t_1, t_2, \ldots , t_r\}$, then its complement ${\overline{h}}$ is also associated with the same composition structure (see Supplementary Information, Property 1). Figure 1b provides a schematic illustration of a composed BF belonging to the composition structure $\{1,2\}$. Fink and Hannam^47^ have provided exact analytical expressions for the number of composed BFs in a composition structure. Following these analytical expressions, it can be easily shown that the composed BFs belonging to the two composition structures $\{1,1,1,\ \ldots ,\ 1\}$ and $\{k\}$ do not restrict the space of *k*-input BFs, and they each include all $2^{2^k}$ possible BFs (see Supplementary Information, Property 2). Thus, for *k*-input BFs, these two composition structures can be considered as trivial whereas the remaining composition structures are in fact non-trivial. Further, it is easy to see that there are no non-trivial composition structures for 1-input and 2-input BFs. Importantly, we excluded all the trivial composition structures from the analyses reported in this work, and in particular, we focus on non-trivial composition structures corresponding to 3, 4 and 5 input BFs.

### Accounting for all the permutations of inputs of composed BFs

Consider a composed BF of the type $g(p_1(x_1), p_2(x_2,x_3))$ that belongs to the composition structure $\{1,2\}$ and corresponds to a 3-input BF $h(x_1,x_2,x_3)$. Taking $p_1(x_1)=x_1$, $p_2(x_2,x_3)=x_2x_3$, and $g(x,y)=x+y$ leads to the composed BF $h(x_1,x_2,x_3) = x_1 + x_2x_3$. However the BFs obtained by permuting the labels of these variables, namely $x_2 + x_1x_3$ and $x_3 + x_1x_2$, are just as relevant biologically; indeed, the labels point to genes and these are hardly ever equivalent. Thus, we count all three of the cases above as valid composed BFs. In contrast, Fink and Hannam^47^ count them as one composed BF. A code to generate all the composed BFs for any given composition structure after accounting for all the permutations of the input variables is available from the associated GitHub repository (see https://github.com/asamallab/CoSt). Note that this example shows that the two ways of counting are not generally related by the number of permutations (*k*!) of *k* labels because of possible symmetries within these expressions.

### TF binding regions and active enhancers

We relied on two types of published datasets for estimating the prevalence of composition structures arising from cis-regulatory modules: (i) transcription factor binding regions and (ii) active enhancers. We focused on the two well-studied human cell lines HepG2 and K562 because there is ample published data for them. We obtained the DNA binding regions of the TFs as ChIP-seq narrowPeak bed files for the two cell lines from the human ENCODE project^78^. The active enhancers are obtained from data processed using the STARRPeaker peak-calling software^79^. Employing these two datasets, we consider that a TF binds to an active enhancer if and only if both the midpoint and the summit of the ChIP-seq peaks for that TF fall within the active enhancer region. Notably, there were no cases wherein the summit of the peaks were not provided in the ChIP-seq files obtained from the human ENCODE project. The ChIP-seq narrowPeak bed files for the HepG2 and K562 cell lines were last downloaded on April $28{\text {th}}$ 2022 and April $29{\text {th}}$ 2022, respectively, from the human ENCODE project website: https://www.encodeproject.org. The processed datasets from human ENCODE used for this analysis and the associated codes are available at: https://github.com/asamallab/CoSt. This study was carried out in accordance with relevant guidelines and regulations.

### Reference biological dataset of 2687 Boolean functions from reconstructed models

We utilized the empirical dataset compiled by Subbaroyan et al.^38^ consisting of 2687 BFs extracted from 88 published discrete models of biological systems to quantify the abundance of composed BFs in biological networks. This dataset of BFs, available for download at https://github.com/asamallab/MCBF/tree/main/biological_dataset, was compiled using information on published models in online repositories Cell Collective^31^ (https://cellcollective.org/), GINSIM^28^ (http://ginsim.org/) or BioModels^29^ (http://www.ebi.ac.uk/biomodels/) and by manually retrieving information from published literature. The compiled collection of 88 published models spans a diverse range of biological processes from various kingdoms of life. Though most of the 88 models are Boolean, some models are not Boolean but nevertheless discrete, implying that each node *may* have more than 2 states in its discrete logic model. For such models, BFs alone were compiled into the dataset, i.e., nodes which had a binary state and whose inputs were restricted to binary states were included in the 2687 BFs.

### Statistical tests

#### Enrichments and relative enrichments

For a given number of inputs *k*, let *T* be the set of composed BFs allowed by the composition structure $\{t_1, t_2, \ldots , t_r\}$, with $t_1 +t_2 + \ldots + t_r = k$. Let $f_0$ be the fraction occupied by the set *T* among the set of all *k*-input BFs. This fraction $f_0$ is equal to the probability of obtaining a BF belonging to the set *T* when drawing at random (uniformly) within all *k*-input BFs (black dots in Fig. 4a). Let $f_1$ denote the fraction of BFs belonging to *T* among all *k*-input BFs that are present in the reference biological dataset (bar plots in Fig. 4a). *T*’s “enrichment factor”, *E*, is equal to the fraction $f_1/f_0$. An enrichment factor $E > 1$ implies that the composition structure $\{t_1, t_2, \ldots , t_r\}$ is enriched in the reference biological dataset, whereas $E < 1$ implies that the composition structure is depleted.

Consider now a refinement of the previous notion of enrichment to probe the roles of biologically meaningful types. Specifically, let *s* be one of the four types of biologically meaningful BFs (UFs, CFs, NCFs, or RoFs). Denote by $T_s$ the subset of BFs of type *s* in *T*. We then define the “relative enrichment” $E_R$ of type *s* in *T* as $E_R = f_{s,1}/f_{s,0}$. In that ratio, $f_{s,0}$ (respectively $f_{s,1}$) is the fraction of BFs in *T* that belong to $T_s$ (respectively the fraction of BFs in the intersection of *T* and the reference biological dataset that belong to $T_s$). A relative enrichment close to 1 means that an enrichment of $T_s$ is driven by an enrichment of *T* (i.e., by the composition structure) rather than by the biologically meaningful sub-type *s*. In contrast, a large relative enrichment suggests that sub-type *s* is driving enrichment of such BFs in the reference biological dataset even after accounting for enrichment of BFs compatible with a given composition structure.

#### Associated *p* values

We first describe the procedure that we employed to test the significance of an enrichment factor *E* for the set *T* of composed BFs in the composition structure $\{t_1, t_2, \ldots , t_r\}$. A similar statistical test was presented and implemented by Subbaroyan et al.^38^. First, we introduce the null hypothesis, denoted by $H_0$, in which all the *k*-input BFs in the reference biological dataset are drawn from a random ensemble comprised of uniformly distributed *k*-input BFs. The *p* value associated with rejecting this null hypothesis $H_0$ is computed as follows. Recall that $f_0$ is the probability of choosing a *k*-input BF belonging to the set *T* from the random ensemble. Define *M* to be the number of *k*-input BFs in the reference biological dataset. Then, we draw *M* BFs from all *k*-input BFs in the random ensemble, and compute the probability of getting *m* BFs that also belong to the set *T*. This probability is equivalent to getting *m* successes when tossing a biased coin *M* times, and is thus given by the binomial distribution $$\left( {\begin{array}{c}M\\ m\end{array}}\right) f_0^m (1-f_0)^{M-m}$$. The *p* value is then given by $$\sum _{m >= M f_1}\left( {\begin{array}{c}M\\ m\end{array}}\right) f_0^m (1-f_0)^{M-m}$$. Here, $M f_1$ is the number of BFs belonging to the set *T* among all *k*-input BFs that are present in the reference biological dataset.

We perform a similar statistical test to determine whether a relative enrichment $E_R$ for a given *T* and *s* is statistically significant. In this case, the null hypothesis $H_0$ hypothesizes that although there is a selection for *T* (as evident from a large value of *E*), the elements that are drawn within *T* have a uniform probability, that is the individual elements belonging to $T_s$ are not more probable than the other elements of *T*. In practice, we draw a sample of size *M* under $H_0$ where as above *M* is the number of *k*-input BFs in the reference biological dataset. If this sample contains $M_T$ elements in *T* as does the reference biological dataset, the distribution of the number of elements in $T_s$ is then known. Specifically, the probability to have *m* elements in $T_s$ is $$\left( {\begin{array}{c}M_T\\ m\end{array}}\right) f_{s,0}^m (1-f_{s,0})^{M_T-m}$$ where $f_{s,0}$ is the ratio of sizes of $T_s$ and *T* in the random ensemble. The *p* value associated with rejecting $H_0$ is then just the sum of all such probabilities under the condition that *m* is larger or equal to the number of $T_s$ elements in the reference biological dataset.

## Supplementary Information

Supplementary Information.

## Data Availability

The list of human macromolecular complexes was abtained from the EBI Complex Portal database^57^, and the list of human TFs was obtained from http://humantfs.ccbr.utoronto.ca/^58^. The families of human TFs are provided in the JASPAR database^64^. The ChIP-seq peaks for TFs in HepG2 and K562 were obtained obtained the human ENCODE project^78^. The active enhancer regions were obtained from data processed using STARRPeaker peak-calling software^79^.

## Code availability

All original codes used to generate the composed BFs are deposited in GitHub and are publically available at https://github.com/asamallab/CoSt.

## References

[1] Chen K, Rajewsky N (2007). The evolution of gene regulation by transcription factors and microRNAs. Nat. Rev. Genet..

[2] Milo R (2002). Network motifs: Simple building blocks of complex networks. Science.

[3] Barabási AL, Oltvai ZN (2004). Network biology: Understanding the cell’s functional organization. Nat. Rev. Genet..

[4] Bornholdt S (2005). Less is more in modeling large genetic networks. Science.

[5] Alon U (2006). An Introduction to Systems Biology: Design Principles of Biological Circuits.

[6] Kauffman SA (1969). Metabolic stability and epigenesis in randomly constructed genetic nets. J. Theor. Biol..

[7] Kauffman SA (1969). Homeostasis and differentiation in random genetic control networks. Nature.

[8] Thomas R (1973). Boolean formalization of genetic control circuits. J. Theor. Biol..

[9] Thomas, R. *Kinetic logic: a Boolean approach to the analysis of complex regulatory systems, Proceedings of the EMBO course “Formal analysis of genetic regulation”, held in Brussels, September 6–16, 1977, Lecture notes in Biomathematics*, vol. 29 (Springer, 1979).

[10] Kauffman SA, Peterson C, Samuelsson B, Troein C (2003). Random Boolean network models and the yeast transcriptional network. Proc. Natl. Acad. Sci..

[11] Villani M, Barbieri A, Serra R (2011). A dynamical model of genetic networks for cell differentiation. PLoS ONE.

[12] Davidich M, Bornholdt S (2008). The transition from differential equations to Boolean networks: A case study in simplifying a regulatory network model. J. Theor. Biol..

[13] Li S, Assmann SM, Albert R (2006). Predicting essential components of signal transduction networks: A dynamic model of guard cell abscisic acid signaling. PLoS Biol..

[14] Saez-Rodriguez J (2007). A logical model provides insights into T cell receptor signaling. PLoS Comput. Biol..

[15] Samal, A. & Jain, S. The regulatory network of *E. coli* metabolism as a Boolean dynamical system exhibits both homeostasis and flexibility of response. *BMC Systems Biology* **2**, 1–18 (2008). 10.1186/1752-0509-2-21 PMC2322946 18312613

[16] Bauer AL, Jackson TL, Jiang Y, Rohlf T (2010). Receptor cross-talk in angiogenesis: mapping environmental cues to cell phenotype using a stochastic, Boolean signaling network model. J. Theor. Biol..

[17] Shmulevich I, Kauffman SA (2004). Activities and sensitivities in Boolean network models. Phys. Rev. Lett..

[18] Drossel B, Mihaljev T, Greil F (2005). Number and length of attractors in a critical Kauffman model with connectivity one. Phys. Rev. Lett..

[19] Klemm K, Bornholdt S (2005). Stable and unstable attractors in Boolean networks. Phys. Rev. E.

[20] Palsson BØ (2006). Systems Biology: Properties of Reconstructed Networks.

[21] Pandey S (2010). Boolean modeling of transcriptome data reveals novel modes of heterotrimeric G-protein action. Mol. Syst. Biol..

[22] Nykter M (2008). Gene expression dynamics in the macrophage exhibit criticality. Proc. Natl. Acad. Sci..

[23] Balleza E (2008). Critical dynamics in genetic regulatory networks: examples from four kingdoms. PLoS ONE.

[24] Chowdhury S (2010). Information propagation within the genetic network of Saccharomyces cerevisiae. BMC Syst. Biol..

[25] Daniels BC (2018). Criticality distinguishes the ensemble of biological regulatory networks. Phys. Rev. Lett..

[26] Mendoza L, Thieffry D, Alvarez-Buylla ER (1999). Genetic control of flower morphogenesis in Arabidopsis thaliana: a logical analysis. Bioinformatics.

[27] Albert R, Othmer HG (2003). The topology of the regulatory interactions predicts the expression pattern of the segment polarity genes in Drosophila melanogaster. J. Theor. Biol..

[28] Gonzalez AG, Naldi A, Sanchez L, Thieffry D, Chaouiya C (2006). GINsim: A software suite for the qualitative modelling, simulation and analysis of regulatory networks. Biosystems.

[29] Li C (2010). BioModels database: An enhanced, curated and annotated resource for published quantitative kinetic models. BMC Syst. Biol..

[30] Saadatpour A (2011). Dynamical and structural analysis of a T cell survival network identifies novel candidate therapeutic targets for large granular lymphocyte leukemia. PLoS Comput. Biol..

[31] Helikar T (2012). The Cell Collective: toward an open and collaborative approach to systems biology. BMC Syst. Biol..

[32] Méndez A, Mendoza L (2016). A network model to describe the terminal differentiation of B cells. PLoS Comput. Biol..

[33] Guberman E, Sherief H, Regan ER (2020). Boolean model of anchorage dependence and contact inhibition points to coordinated inhibition but semi-independent induction of proliferation and migration. Comput. Struct. Biotechnol. J..

[34] Aracena J (2008). Maximum number of fixed points in regulatory Boolean networks. Bull. Math. Biol..

[35] Kauffman SA (1993). The Origins of Order: Self-Organization and Selection in Evolution.

[36] Jarrah AS, Raposa B, Laubenbacher R (2007). Nested canalyzing, unate cascade, and polynomial functions. Phys. D.

[37] Kadelka C, Kuipers J, Laubenbacher R (2017). The influence of canalization on the robustness of Boolean networks. Phys. D.

[38] Subbaroyan A, Martin OC, Samal A (2022). Minimum complexity drives regulatory logic in boolean models of living systems. PNAS Nexus.

[39] Graudenzi A, Serra R, Villani M, Damiani C, Colacci A, Kauffman S (2011). Dynamical properties of a Boolean model of gene regulatory network with memory. J. Comput. Biol..

[40] Graudenzi A, Serra R, Villani M, Colacci A, Kauffman S (2011). Robustness analysis of a Boolean model of gene regulatory network with memory. J. Comput. Biol..

[41] Schwanhäusser B (2011). Global quantification of mammalian gene expression control. Nature.

[42] Flöttmann M, Krause F, Klipp E, Krantz M (2013). Reaction-contingency based bipartite Boolean modelling. BMC Syst. Biol..

[43] Mori T, Flöttmann M, Krantz M, Akutsu T, Klipp E (2015). Stochastic simulation of Boolean rxncon models: towards quantitative analysis of large signaling networks. BMC Syst. Biol..

[44] Hannam R, Kühn R, Annibale A (2019). Percolation in bipartite Boolean networks and its role in sustaining life. J. Phys. A Math. Theor..

[45] Torrisi G, Kühn R, Annibale A (2020). Percolation on the gene regulatory network. J. Stat. Mech. Theory Exp..

[46] Rottensteiner H, Kal AJ, Hamilton B, Ruis H, Tabak HF (1997). A heterodimer of the Zn2Cys6 transcription factors Pip2p and Oaf1p controls induction of genes encoding peroxisomal proteins in Saccharomyces cerevisiae. Eur. J. Biochem..

[47] Fink, T. & Hannam, R. Boolean composition restricts biological logics. arXiv preprint arXiv:2109.12551 (2021).

[48] Shmulevich I, Lähdesmäki H, Dougherty ER, Astola J, Zhang W (2003). The role of certain post classes in Boolean network models of genetic networks. Proc. Natl. Acad. Sci..

[49] Montagna S, Braccini M, Roli A (2020). The impact of self-loops on Boolean networks attractor landscape and implications for cell differentiation modelling. IEEE/ACM Trans. Comput. Biol. Bioinform..

[50] Shlyueva D, Stampfel G, Stark A (2014). Transcriptional enhancers: from properties to genome-wide predictions. Nat. Rev. Genet..

[51] Reiter F, Wienerroither S, Stark A (2017). Combinatorial function of transcription factors and cofactors. Curr. Opin. Genet. Dev..

[52] Fernandes L, Rodrigues-Pousada C, Struhl K (1997). Yap, a novel family of eight bZIP proteins in Saccharomyces cerevisiae with distinct biological functions. Mol. Cell. Biol..

[53] Wolberger C (1999). Multiprotein-DNA complexes in transcriptional regulation. Annu. Rev. Biophys. Biomol. Struct..

[54] Vernoux T (2011). The auxin signalling network translates dynamic input into robust patterning at the shoot apex. Mol. Syst. Biol..

[55] Funnell, A. P. W. & Crossley, M. Homo- and heterodimerization in transcriptional regulation. In *Protein Dimerization and Oligomerization in Biology*, vol. 747, 105–121 (Springer New York, New York, NY, 2012). 10.1007/978-1-4614-3229-6_7 22949114

[56] Guilfoyle TJ, Hagen G (2007). Auxin response factors. Curr. Opin. Plant Biol..

[57] Meldal BHM (2022). Complex Portal 2022: new curation frontiers. Nucleic Acids Res..

[58] Lambert SA (2018). The human transcription factors. Cell.

[59] Consortium & T. U. UniProt: the universal protein knowledgebase in 2021. *Nucleic Acids Res.* **49**, D480–D489 (2020). 10.1093/nar/gkaa1100 PMC7778908 33237286

[60] Dröge-Laser W, Snoek BL, Snel B, Weiste C (2018). The Arabidopsis bZIP transcription factor family—an update. Curr. Opin. Plant Biol..

[61] Chen L, Lopes JM (2010). Multiple bHLH proteins regulate CIT2 expression in Saccharomyces cerevisiae. Yeast.

[62] Rodríiguez-Martínez JA, Reinke AW, Bhimsaria D, Keating AE, Ansari AZ (2017). Combinatorial bZIP dimers display complex DNA-binding specificity landscapes. eLife.

[63] Jones S (2004). An overview of the basic helix-loop-helix proteins. Genome Biol..

[64] Castro-Mondragon JA (2021). JASPAR 2022: the 9th release of the open-access database of transcription factor binding profiles. Nucleic Acids Res..

[65] Spitz F, Furlong EE (2012). Transcription factors: from enhancer binding to developmental control. Nat. Rev. Genet..

[66] Sandelin A (2007). Mammalian RNA polymerase II core promoters: insights from genome-wide studies. Nat. Rev. Genet..

[67] Blackwood EM, Kadonaga JT (1998). Going the distance: a current view of enhancer action. Science.

[68] Rao S, Ahmad K, Ramachandran S (2021). Cooperative binding between distant transcription factors is a hallmark of active enhancers. Mol. Cell.

[69] Lex A, Gehlenborg N, Strobelt H, Vuillemot R, Pfister H (2014). UpSet: Visualization of Intersecting Sets. IEEE Trans. Vis. Comput. Graphics.

[70] Nikolajewa S, Friedel M, Wilhelm T (2007). Boolean networks with biologically relevant rules show ordered behavior. Biosystems.

[71] Wang R, Albert R (2011). Elementary signaling modes predict the essentiality of signal transduction network components. BMC Syst. Biol..

[72] Zañudo JGT, Albert R (2013). An effective network reduction approach to find the dynamical repertoire of discrete dynamic networks. Chaos Interdiscip. J. Nonlinear Sci..

[73] Gates AJ, Brattig Correia R, Wang X, Rocha L (2021). The effective graph reveals redundancy, canalization, and control pathways in biochemical regulation and signaling. Proc. Natl. Acad. Sci..

[74] Thomas R (1991). Regulatory networks seen as asynchronous automata: a logical description. J. Theor. Biol..

[75] Feldman J (2003). A catalog of Boolean concepts. J. Math. Psychol..

[76] Szallasi, Z. & Liang, S. Modeling the normal and neoplastic cell cycle with ‘realistic Boolean genetic networks’: Their application for understanding carcinogenesis and assessing therapeutic strategies. In *Pacific Symposium on Biocomputing*, vol. 3, 66–76 (Citeseer, 1998). 9697172

[77] Golumbic, M. C., Gurvich, V., Crama, Y. & Hammer, P. L. *Read-once functions. Encyclopedia of Mathematics and its Applications* 448–486 (Cambridge University Press, Cambridge, 2011).

[78] Consortium *et al.* An integrated encyclopedia of DNA elements in the human genome. *Nature ***489**, 57 (2012). 10.1038/nature11247 PMC3439153 22955616

[79] Lee D (2020). STARRPeaker: uniform processing and accurate identification of STARR-seq active regions. Genome Biol..

