# Supplementary material for: Relative importance of composition structures and biologically meaningful logics in bipartite Boolean models of gene regulation
Source: Sci Rep. 2022 Oct 28;12:18156. doi: 10.1038/s41598-022-22654-7 (PMC9616893; doi:10.1038/s41598-022-22654-7)
Supplement: Supplementary file 1 — Supplementary Information. [file 41598_2022_22654_MOESM1_ESM.pdf]

Supplementary Information for Relative importance of composition structures and biologically meaningful logics in bipartite Boolean models of gene regulation

Yasharth Yadav[1,+], Ajay Subbaroyan[1,2,+], Olivier C. Martin[3,4,*], and Areejit Samal[1,2,*]

[1]The Institute of Mathematical Sciences (IMSc), Chennai 600113, India
[2]Homi Bhabha National Institute (HBNI), Mumbai 400094, India
[3]Université Paris-Saclay, CNRS, INRAE, Univ Evry, Institute of Plant Sciences Paris-Saclay (IPS2), 91190 Gif sur Yvette, France
[4]Université Paris Cité, CNRS, INRAE, Institute of Plant Sciences Paris-Saclay (IPS2), 91190 Gif sur Yvette, France
[+]Y.Y. and Aj.S. contributed equally to this work and should be considered as Joint-First authors
[*]To whom correspondence should be addressed: asamal@imsc.res.in or olivier.c.martin@inrae.fr

Complexes in Yeast

To perform the empirical analysis in *S. cerevisiae*, we first obtained a list of 617 macromolecular complexes in *S. cerevisiae* from the EBI Complex portal database[1]. Then we obtained the list of TFs in *S. cerevisiae* from the Yeastract database[2]. To do this, we obtained a list of 5195 verified genes from the SGD YeastMine database[3], and provided these genes as input to the Yeastract database. Additionally, we used the query "DNA binding evidence or expression evidence" in the Yeastract database. "DNA binding evidence" includes TF regulation verified by experiments such as EMSA, ChIP, ChIP-chip and ChIP-seq, DNA footprinting, whereas "expression evidence" includes those interactions established by comparing gene expression in wild-type strains with mutant strains in which the gene encoding the TF is mutated. Expression evidence is obtained via northern blotting, RT-PCR, DNA microarrays and RNA-seq experiments[2]. The above query in the Yeastract database resulted in a list of 217 TFs that were used for further analysis. Note that 153 out of these 217 TFs show evidence for both DNA binding and effects on expression. Finally, among the 617 macromolecular complexes we selected only those complexes in which all the protein subunits correspond to TFs.

Using the EBI complex portal and the TFs from Yeastract database, we found that there are 17 heteromeric complexes among the 617 complexes in *S. cerevisiae* such that each of their protein subunits correspond to TFs (see Supplementary Table S3). Thereafter, we ascertained via manual curation of the literature associated with these 17 complexes that 15 of them act as TRs, 1 of them binds to DNA but whether it regulates gene expression is uncertain, and the remaining 1 acts as a transcriptional co-repressor. Among the 15 complexes that act as TRs, 9 complexes are formed by 2 proteins, 5 complexes are formed by 3 proteins and 1 complex is formed by 4 proteins. There are 30 unique TFs whose combination results in these 15 complexes. Additionally, we found that 11 out of these 15 complexes are such that their protein subunits show both DNA binding and expression evidence.

It is known from experiments that TFs belonging to the basic leucine zipper factors (bZIP)[4,5] and basic helix-loop-helix factors (bHLH)[6,7] classes typically bind DNA as dimers. To determine the classes of the 217 TFs in *S. cerevisiae*, we utilized the JASPAR database[8]. In *S. cerevisiae*, we found 10 TFs that belong to the bZIP class and 7 TFs that belong to the bHLH class (see Supplementary Table S4). Further, we find that all the 17 TFs in the bZIP or bHLH classes display evidence for both DNA binding and effects on expression.

Properties of composed Boolean Functions

Property 1. Given a composition structure $\{t_1, t_2, \ldots, t_r\}$, if h is a possible composed BF, then its complement $\overline{h}$ is also a possible composed BF.
Proof: h is a composed BF of the form $g(p_1, p_2, \ldots, p_r)$, where each p_i is a BF of t_i inputs. There are $2^{2^{t_1}} 2^{2^{t_2}} \ldots 2^{2^{t_r}} 2^{2^r}$ combi-

nations of $p_1, p_2, \ldots, p_r$ and g which comprise the composed BFs h. Let us consider one such combination $p_1^*, p_2^*, \ldots, p_r^*$ and g^*, that corresponds to a BF h^* from the space of all composed BFs. Now, there also exists another combination $p_1^*, p_2^*, \ldots, p_r^*$ and $\overline{g^*}$ among the $2^{2^{t_1}} 2^{2^{t_2}} \ldots 2^{2^{t_r}} 2^{2^r}$ combinations, which corresponds to the BF $\overline{h^*}$. Hence, a function and its complement are both present in the composed BFs of any composition structure.

Property 2. The composition structure $\{k\}$ does not restrict the space of BFs.
Proof: The composition structure $\{k\}$ corresponds to a k-input BF of the form $h = g(p_1(x_1, x_2, \ldots, x_k))$. There are 2^{2^k} BFs that can be assigned to p_1, and $2^{2^1} = 4$ BFs that can be assigned to g. Among the 4 BFs that can be assigned to g, if we consider the $g(p_1) = p_1$, then $h = p_1(x_1, x_2, \ldots, x_k)$. It follows that any BF among the 2^{2^k} possible k-input BFs can be assigned to h. Since h spans all the possible k-input BFs, the composition structure $\{k\}$ cannot restrict the space of BFs.

References

1. Meldal, B. H. M. *et al.* Complex Portal 2022: new curation frontiers. *Nucleic Acids Res.* **50**, D578–D586 (2022).
2. Teixeira, M. C. *et al.* Yeastract: an upgraded database for the analysis of transcription regulatory networks in *Saccharomyces cerevisiae*. *Nucleic Acids Res.* **46**, D348–D353 (2018).
3. Balakrishnan, R. *et al.* Yeastmine—an integrated data warehouse for *Saccharomyces cerevisiae* data as a multipurpose tool-kit. *Database* **2012** (2012).
4. Rodríguez-Martínez, J. A., Reinke, A. W., Bhimsaria, D., Keating, A. E. & Ansari, A. Z. Combinatorial bZIP dimers display complex DNA-binding specificity landscapes. *eLife* **6**, e19272 (2017).
5. Dröge-Laser, W., Snoek, B. L., Snel, B. & Weiste, C. The Arabidopsis bZIP transcription factor family — an update. *Curr. Opin. Plant Biol.* **45**, 36–49 (2018).
6. Jones, S. An overview of the basic helix-loop-helix proteins. *Genome Biol.* **5**, 6 (2004).
7. Chen, L. & Lopes, J. M. Multiple bHLH proteins regulate CIT2 expression in *Saccharomyces cerevisiae*. *Yeast* **27**, 345–359 (2010).
8. Castro-Mondragon, J. A. *et al.* JASPAR 2022: the 9th release of the open-access database of transcription factor binding profiles. *Nucleic Acids Res.* **50**, D165–D173 (2021).

Supplementary figures

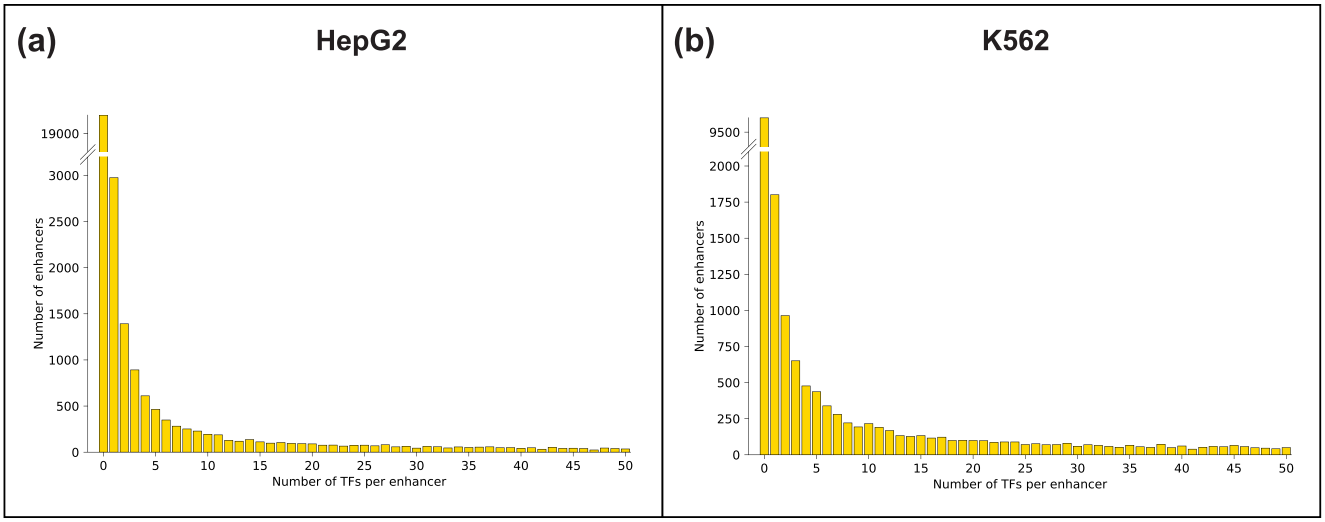

Figure S1. Prevalance of active enhancers bound by multiple TFs in human cell lines. (a) Histogram showing the number of active enhancers bound by a given number of TFs in the HepG2 cell line in humans. We found that 32.68% of the active enhancers in HepG2 are bound by at least 2 TFs. (b) Histogram showing the number of active enhancers bound by a given number of TFs in the K562 cell line in humans. We found that 44.31% of the active enhancers in K562 are bound by at least 2 TFs. These results suggest that non-trivial composition structures are prevalent in gene regulatory networks.

Supplementary tables

Table S1. A list of 169 complexes from the set of 1325 complexes in *H. sapiens* such that all the protein subunits of these complexes are transcription factors. The 1325 complexes in *H. sapiens* were obtained from the EBI Complex Portal database. The list of human TFs was obtained from `http://humantfs.ccbr.utoronto.ca/`. 'Complex ID' is the identifier of the complex as given in the EBI Complex Portal database. 'Complex name' is the name of the complex as given in the EBI Complex Portal database. 'Size of the complex' is the number of protein subunits the complex is constituted of. 'Uniprot ID of protein subunits' gives the Uniprot IDs of the subunits in a complex and their stoichiometric coefficients as integers within brackets. Stoichiometric coefficients which are unknown are represented by (−). 'Is a TR' column is 'yes' if the complex acts as a transcriptional regulator (TR) based on manual literature curation.

Complex ID	Complex name	Size of the complex	UniProt ID of protein subunits	Is a TR
CPX-6405	bZIP transcription factor complex, ATF1-CREB1	2	P16220(1)<br>P18846(1)	yes
CPX-6414	bZIP transcription factor complex, ATF2-BATF3	2	P15336(1)<br>Q9NR55(1)	yes
CPX-6416	bZIP transcription factor complex, ATF2-FOS	2	P01100(1)<br>P15336(1)	yes
CPX-6420	bZIP transcription factor complex, ATF2-JUN	2	P05412(1)<br>P15336(1)	yes
CPX-6421	bZIP transcription factor complex, ATF2-JUNB	2	P15336(1)<br>P17275(1)	yes
CPX-6467	bZIP transcription factor complex, ATF3-BATF	2	P18847(1)<br>Q16520(1)	yes
CPX-6468	bZIP transcription factor complex, ATF3-BATF3	2	P18847(1)<br>Q9NR55(1)	yes
CPX-6469	bZIP transcription factor complex, ATF3-CEBPA	2	P18847(1)<br>P49715(1)	yes
CPX-6471	bZIP transcription factor complex, ATF3-CEBPG	2	P17676(1)<br>P18847(1)	yes
CPX-6477	bZIP transcription factor complex, ATF3-FOS	2	P01100(1)<br>P18847(1)	yes
CPX-6478	bZIP transcription factor complex, ATF3-FOSL1	2	P15407(1)<br>P18847(1)	yes
CPX-6474	bZIP transcription factor complex, ATF3-JUN	2	P05412(1)<br>P18847(1)	yes
CPX-6476	bZIP transcription factor complex, ATF3-JUNB	2	P17275(1)<br>P18847(1)	yes
CPX-6523	bZIP transcription factor complex, ATF4-BATF2	2	P18848(1)<br>Q8N1L9(1)	yes

CPX-6524	bZIP transcription factor complex, ATF4-BATF3	2	P18848(1)<br>Q9NR55(1)	yes
CPX-6525	bZIP transcription factor complex, ATF4-CEBPA	2	P18848(1)<br>P49715(1)	yes
CPX-6527	bZIP transcription factor complex, ATF4-CEBPG	2	P18848(1)<br>P53567(1)	yes
CPX-6563	bZIP transcription factor complex, ATF4-JUNB	2	P17275(1)<br>P18848(1)	yes
CPX-6567	bZIP transcription factor complex, ATF4-MAFB	2	P18848(1)<br>Q9Y5Q3(1)	yes
CPX-6585	bZIP transcription factor complex, ATF5-BATF	2	Q16520(1)<br>Q9Y2D1(1)	yes
CPX-6586	bZIP transcription factor complex, ATF5-CEBPA	2	P49715(1)<br>Q9Y2D1(1)	yes
CPX-6589	bZIP transcription factor complex, ATF5-CEBPE	2	Q15744(1)<br>Q9Y2D1(1)	yes
CPX-6588	bZIP transcription factor complex, ATF5-CEBPG	2	P53567(1)<br>Q9Y2D1(1)	yes
CPX-7006	bZIP transcription factor complex, BATF-CEBPA	2	P49715(1)<br>Q16520(1)	yes
CPX-7010	bZIP transcription factor complex, BATF-CEBPE	2	Q15744(1)<br>Q16520(1)	yes
CPX-7008	bZIP transcription factor complex, BATF-CEBPG	2	P53567(1)<br>Q16520(1)	yes
CPX-7011	bZIP transcription factor complex, BATF-HLF	2	Q16520(1)<br>Q16534(1)	yes
CPX-7003	bZIP transcription factor complex, BATF-JUNB	2	P17275(1)<br>Q16520(1)	yes
CPX-7017	bZIP transcription factor complex, BATF-NFIL3	2	Q16520(1)<br>Q16649(1)	yes
CPX-7063	bZIP transcription factor complex, BATF2-JUN	2	P05412(1)<br>Q8N1L9(1)	yes
CPX-7061	bZIP transcription factor complex, BATF2-JUNB	2	P17275(1)<br>Q8N1L9(1)	yes
CPX-7095	bZIP transcription factor complex, BATF3-CEBPA	2	P49715(1)<br>Q9NR55(1)	yes
CPX-7097	bZIP transcription factor complex, BATF3-CEBPG	2	P53567(1)<br>Q9NR55(1)	yes

CPX-7100	bZIP transcription factor complex, BATF3-JUN	2	P05412(1)<br>Q9NR55(1)	yes
CPX-7101	bZIP transcription factor complex, BATF3-JUNB	2	P17275(1)<br>Q9NR55(1)	yes
CPX-486	bZIP transcription factor complex, FOS-JUN	2	P01100(1)<br>P05412(1)	yes
CPX-504	c-Myb-C/EBPbeta complex	2	P10242(1)<br>P17676(2)	yes
CPX-1956	CCAAT-binding factor complex	3	P23511(1)<br>P25208(1)<br>Q13952(1)	yes
CPX-3229	CLOCK-BMAL1 transcription complex	2	O00327(1)<br>O15516(1)	yes
CPX-3230	CLOCK-BMAL2 transcription complex	2	O15516(1)<br>Q8WYA1(1)	yes
CPX-5156	ERalpha-NCOA2 activated estrogen receptor complex	2	P03372(2)<br>Q15596(2)	yes
CPX-1123	FOXO3-MYC complex	2	O43524(-)<br>P01106(-)	yes
CPX-6016	ISGF3 complex	3	P42224(-)<br>P52630(-)<br>Q00978(-)	yes
CPX-5834	NF-kappaB DNA-binding transcription factor complex, p65/c-Rel	2	Q04206(1)<br>Q04864(1)	yes
CPX-517	PXR-NCOA1 activated nuclear receptor complex	2	O75469(2)<br>Q15788(2)	yes
CPX-496	RXRalpha-PXR nuclear receptor complex	2	O75469(2)<br>P19793(2)	yes
CPX-508	RXRalpha-RARalpha retinoic acid receptor complex	2	P10276(1)<br>P19793(1)	yes
CPX-654	RXRalpha-TRbeta nuclear hormone receptor complex	2	P10828(1)<br>P19793(1)	yes
CPX-54	SMAD1-SMAD4 complex	2	Q13485(1)<br>Q15797(2)	yes
CPX-3252	SMAD3-SMAD4 complex	2	P84022(2)<br>Q13485(1)	yes
CPX-6041	STAT1/STAT3 complex	2	P40763(1)<br>P42224(1)	yes

CPX-6042	STAT1/STAT4 complex	2	P42224(1)<br>Q14765(1)	yes
CPX-6043	STAT3/STAT5A complex	2	P40763(1)<br>P42229(1)	yes
CPX-6044	STAT3/STAT5B complex	2	P40763(1)<br>P51692(1)	yes
CPX-91	Transcriptional activator Myc-Max complex	2	P01106(1)<br>P61244(1)	yes
CPX-104	Transcriptional repressor Mad-Max complex	2	P61244(1)<br>Q05195(1)	yes
CPX-3079	USF1-USF2 upstream stimulatory factor complex	2	P22415(1)<br>Q15853(1)	yes
CPX-6419	bZIP transcription factor complex, ATF2-JDP2	2	P15336(1)<br>Q8WYK2(1)	yes
CPX-6422	bZIP transcription factor complex, ATF2-JUND	2	P15336(1)<br>P17535(1)	yes
CPX-6595	bZIP transcription factor complex, ATF6-ATF6B	2	P18850(1)<br>Q99941(1)	yes
CPX-6600	bZIP transcription factor complex, ATF6B-XBP1	2	P17861(1)<br>Q99941(1)	yes
CPX-2500	bZIP transcription factor complex, BACH1-MAF	2	O14867(1)<br>O75444(1)	yes
CPX-7165	bZIP transcription factor complex, BACH1-MAFF	2	O14867(1)<br>Q9ULX9(1)	yes
CPX-2872	bZIP transcription factor complex, BACH1-MAFG	2	O14867(1)<br>O15525(1)	yes
CPX-2493	bZIP transcription factor complex, BACH1-MAFK	2	O14867(1)<br>O60675(1)	yes
CPX-2483	bZIP transcription factor complex, BACH2-MAF	2	O75444(1)<br>Q9BYV9(1)	yes
CPX-2484	bZIP transcription factor complex, BACH2-MAFF	2	Q9BYV9(1)<br>Q9ULX9(1)	yes
CPX-2482	bZIP transcription factor complex, BACH2-MAFK	2	O60675(1)<br>Q9BYV9(1)	yes
CPX-7005	bZIP transcription factor complex, BATF-JUN	2	P05412(1)<br>Q16520(1)	yes
CPX-7102	bZIP transcription factor complex, BATF3-JUND	2	P17535(1)<br>Q9NR55(1)	yes

CPX-509	bZIP transcription factor complex, CEBPA-CEBPB	2	P17676(1)<br>P49715(1)	yes
CPX-1971	E2F1-DP1 transcription factor complex	2	Q01094(1)<br>Q14186(1)	yes
CPX-1972	E2F2-DP1 transcription factor complex	2	Q14186(1)<br>Q14209(1)	yes
CPX-711	PPARgamma-NCOA1 activated nuclear receptor complex	2	P37231(1)<br>Q15788(1)	yes
CPX-702	PPARgamma-NCOA2 activated nuclear receptor complex	2	P37231(1)<br>Q15596(1)	yes
CPX-525	RARalpha-NCOA1 activated retinoic acid receptor complex	2	P10276(2)<br>Q15788(2)	yes
CPX-666	RARalpha-NCOA2 activated retinoic acid receptor complex	2	P10276(2)<br>Q15596(2)	yes
CPX-632	RXRalpha-LXRalpha nuclear hormone receptor complex	2	P19793(1)<br>Q13133(1)	yes
CPX-678	RXRalpha-LXRbeta nuclear hormone receptor complex	2	P19793(1)<br>P55055(1)	yes
CPX-513	RXRalpha-NCOA2 activated retinoic acid receptor complex	2	P19793(2)<br>Q15596(2)	yes
CPX-816	RXRalpha-RARalpha-NCOA2 retinoic acid receptor complex	3	P10276(1)<br>P19793(1)<br>Q15596(-)	yes
CPX-631	RXRalpha-VDR nuclear hormone receptor complex	2	P11473(1)<br>P19793(1)	yes
CPX-716	RXRbeta-LXRalpha nuclear hormone receptor complex	2	P28702(1)<br>Q13133(1)	yes
CPX-652	RXRbeta-LXRbeta nuclear hormone receptor complex	2	P28702(1)<br>P55055(1)	yes
CPX-871	RXRbeta-VDR nuclear hormone receptor complex	2	P11473(1)<br>P28702(1)	yes
CPX-6062	SMAD3-TTF-1 complex	2	P43699(-)<br>P84022(-)	yes
CPX-2497	bZIP transcription factor complex, BACH1-MAFB	2	O14867(1)<br>Q9Y5Q3(1)	uncertain
CPX-6407	bZIP transcription factor complex, ATF2-ATF3	2	P15336(1)<br>P18847(1)	uncertain

CPX-6408	bZIP transcription factor complex, ATF2-ATF4	2	P15336(1)<br>P18848(1)	uncertain
CPX-6415	bZIP transcription factor complex, ATF2-DDIT3	2	P15336(1)<br>P35638(1)	uncertain
CPX-6417	bZIP transcription factor complex, ATF2-FOSL1	2	P15336(1)<br>P15407(1)	uncertain
CPX-6385	bZIP transcription factor complex, ATF3-ATF4	2	P18847(1)<br>P18848(1)	uncertain
CPX-6472	bZIP transcription factor complex, ATF3-CEBPE	2	P18847(1)<br>Q15744(1)	uncertain
CPX-6473	bZIP transcription factor complex, ATF3-DDIT3	2	P18847(1)<br>P35638(1)	uncertain
CPX-6542	bZIP transcription factor complex, ATF4-CREBZF	2	P18848(1)<br>Q9NS37(1)	uncertain
CPX-6543	bZIP transcription factor complex, ATF4-DDIT3	2	P18848(1)<br>P35638(1)	uncertain
CPX-6564	bZIP transcription factor complex, ATF4-FOS	2	P01100(1)<br>P18848(1)	uncertain
CPX-6565	bZIP transcription factor complex, ATF4-FOSL1	2	P15407(1)<br>P18848(1)	uncertain
CPX-6562	bZIP transcription factor complex, ATF4-JUN	2	P05412(1)<br>P18848(1)	uncertain
CPX-6597	bZIP transcription factor complex, ATF6-XBP1	2	P17861(1)<br>P18850(1)	uncertain
CPX-2485	bZIP transcription factor complex, BACH2-MAFG	2	O15525(1)<br>Q9BYV9(1)	uncertain
CPX-7014	bZIP transcription factor complex, BATF-DBP	2	Q10586(1)<br>Q16520(1)	uncertain
CPX-7004	bZIP transcription factor complex, BATF-DDIT3	2	P35638(1)<br>Q16520(1)	uncertain
CPX-7108	bZIP transcription factor complex, BATF3-DBP	2	Q10586(1)<br>Q9NR55(1)	uncertain
CPX-7106	bZIP transcription factor complex, BATF3-DDIT3	2	P35638(1)<br>Q9NR55(1)	uncertain
CPX-69	bZIP transcription factor complex, CEBPA-DDIT3	2	P35638(1)<br>P49715(1)	uncertain
CPX-70	bZIP transcription factor complex, CEBPB-DDIT3	2	P17676(1)<br>P35638(1)	uncertain

CPX-6047	STAT2/STAT6 complex	2	P42226(1)<br>P52630(1)	uncertain
CPX-6046	STAT3/STAT4 complex	2	P40763(1)<br>Q14765(1)	uncertain
CPX-6045	STAT5A/STAT5B complex	2	P42229(1)<br>P51692(1)	uncertain
CPX-480	AP-1 transcription factor complex FOS-JUN-NFATC2	3	P01100(1)<br>P05412(1)<br>Q13469(1)	uncertain
CPX-9	bZIP transcription factor complex, ATF1-ATF4	2	P18846(1)<br>P18848(1)	uncertain
CPX-6402	bZIP transcription factor complex, ATF1-BACH1	2	O14867(1)<br>P18846(1)	uncertain
CPX-6404	bZIP transcription factor complex, ATF1-NFIL3	2	P18846(1)<br>Q16649(1)	uncertain
CPX-6409	bZIP transcription factor complex, ATF2-ATF7	2	P15336(1)<br>P17544(1)	uncertain
CPX-6412	bZIP transcription factor complex, ATF2-BACH1	2	O14867(1)<br>P15336(1)	uncertain
CPX-6413	bZIP transcription factor complex, ATF2-BATF	2	P15336(1)<br>Q16520(1)	uncertain
CPX-6418	bZIP transcription factor complex, ATF2-FOSL2	2	P15336(1)<br>P15408(1)	uncertain
CPX-6466	bZIP transcription factor complex, ATF3-ATF7	2	P17544(1)<br>P18847(1)	uncertain
CPX-6470	bZIP transcription factor complex, ATF3-CEBPB	2	P17676(1)<br>P18847(1)	uncertain
CPX-6479	bZIP transcription factor complex, ATF3-FOSL2	2	P15408(1)<br>P18847(1)	uncertain
CPX-6480	bZIP transcription factor complex, ATF3-MAFF	2	P18847(1)<br>Q9ULX9(1)	uncertain
CPX-6481	bZIP transcription factor complex, ATF3-MAFG	2	O15525(1)<br>P18847(1)	uncertain
CPX-6522	bZIP transcription factor complex, ATF4-BATF	2	P18848(1)<br>Q16520(1)	uncertain
CPX-6526	bZIP transcription factor complex, ATF4-CEBPB	2	P17676(1)<br>P18848(1)	uncertain

CPX-6528	bZIP transcription factor complex, ATF4-CEBPD	2	P18848(1)<br>P49716(1)	uncertain
CPX-6529	bZIP transcription factor complex, ATF4-CEBPE	2	P18848(1)<br>Q15744(1)	uncertain
CPX-8	bZIP transcription factor complex, ATF4-CREB1	2	P16220(1)<br>P18848(1)	uncertain
CPX-6541	bZIP transcription factor complex, ATF4-CREB3	2	O43889(1)<br>P18848(1)	uncertain
CPX-6566	bZIP transcription factor complex, ATF4-MAF	2	O75444(1)<br>P18848(1)	uncertain
CPX-6568	bZIP transcription factor complex, ATF4-NFE2	2	P18848(1)<br>Q16621(1)	uncertain
CPX-6570	bZIP transcription factor complex, ATF4-NFE2L2	2	P18848(1)<br>Q16236(1)	uncertain
CPX-6572	bZIP transcription factor complex, ATF4-NFE2L3	2	P18848(1)<br>Q9Y4A8(1)	uncertain
CPX-6601	bZIP transcription factor complex, ATF6B-CREBZF	2	Q99941(1)<br>Q9NS37(1)	uncertain
CPX-6781	bZIP transcription factor complex, ATF7-BACH1	2	O14867(1)<br>P17544(1)	uncertain
CPX-6782	bZIP transcription factor complex, ATF7-CEBPG	2	P17544(1)<br>P53567(1)	uncertain
CPX-6784	bZIP transcription factor complex, ATF7-DDIT3	2	P17544(1)<br>P35638(1)	uncertain
CPX-6783	bZIP transcription factor complex, ATF7-FOS	2	P01100(1)<br>P17544(1)	uncertain
CPX-6785	bZIP transcription factor complex, ATF7-FOSL2	2	P15408(1)<br>P17544(1)	uncertain
CPX-6786	bZIP transcription factor complex, ATF7-JUN	2	P05412(1)<br>P17544(1)	uncertain
CPX-6787	bZIP transcription factor complex, ATF7-JUNB	2	P17275(1)<br>P17544(1)	uncertain
CPX-6788	bZIP transcription factor complex, ATF7-JUND	2	P17535(1)<br>P17544(1)	uncertain
CPX-6789	bZIP transcription factor complex, ATF7-NFE2	2	P17544(1)<br>Q16621(1)	uncertain
CPX-7012	bZIP transcription factor complex, BACH1-BATF	2	O14867(1)<br>Q16520(1)	uncertain

CPX-2494	bZIP transcription factor complex, BACH1-CREB1	2	O14867(1)<br>P16220(1)	uncertain
CPX-2496	bZIP transcription factor complex, BACH1-DDIT3	2	O14867(1)<br>P35638(1)	uncertain
CPX-2491	bZIP transcription factor complex, BACH1-FOS	2	O14867(1)<br>P01100(1)	uncertain
CPX-7093	bZIP transcription factor complex, BACH2-BATF3	2	Q9BYV9(1)<br>Q9NR55(1)	uncertain
CPX-2479	bZIP transcription factor complex, BACH2-MAFB	2	Q9BYV9(1)<br>Q9Y5Q3(1)	uncertain
CPX-2471	bZIP transcription factor complex, BACH2-NFE2L3	2	Q9BYV9(1)<br>Q9Y4A8(1)	uncertain
CPX-7018	bZIP transcription factor complex, BATF-BATF3	2	Q16520(1)<br>Q9NR55(1)	uncertain
CPX-7007	bZIP transcription factor complex, BATF-CEBPB	2	P17676(1)<br>Q16520(1)	uncertain
CPX-7009	bZIP transcription factor complex, BATF-CEBPD	2	P49716(1)<br>Q16520(1)	uncertain
CPX-7013	bZIP transcription factor complex, BATF-JUND	2	P17535(1)<br>Q16520(1)	uncertain
CPX-7065	bZIP transcription factor complex, BATF2-CEBPA	2	P49715(1)<br>Q8N1L9(1)	uncertain
CPX-7067	bZIP transcription factor complex, BATF2-CEBPE	2	Q15744(1)<br>Q8N1L9(1)	uncertain
CPX-7066	bZIP transcription factor complex, BATF2-CEBPG	2	P53567(1)<br>Q8N1L9(1)	uncertain
CPX-7068	bZIP transcription factor complex, BATF2-DBP	2	Q10586(1)<br>Q8N1L9(1)	uncertain
CPX-7064	bZIP transcription factor complex, BATF2-DDIT3	2	P35638(1)<br>Q8N1L9(1)	uncertain
CPX-7081	bZIP transcription factor complex, BATF2-HLF	2	Q16534(1)<br>Q8N1L9(1)	uncertain
CPX-7085	bZIP transcription factor complex, BATF2-MAFF	2	Q8N1L9(1)<br>Q9ULX9(1)	uncertain
CPX-7096	bZIP transcription factor complex, BATF3-CEBPB	2	P17676(1)<br>Q9NR55(1)	uncertain
CPX-7098	bZIP transcription factor complex, BATF3-CEBPD	2	P49716(1)<br>Q9NR55(1)	uncertain

CPX-7099	bZIP transcription factor complex, BATF3-CEBPE	2	Q15744(1)<br>Q9NR55(1)	uncertain
CPX-7109	bZIP transcription factor complex, BATF3-CREB3	2	O43889(1)<br>Q9NR55(1)	uncertain
CPX-7107	bZIP transcription factor complex, BATF3-HLF	2	Q16534(1)<br>Q9NR55(1)	uncertain
CPX-7103	bZIP transcription factor complex, BATF3-MAFF	2	Q9NR55(1)<br>Q9ULX9(1)	uncertain
CPX-7105	bZIP transcription factor complex, BATF3-MAFG	2	O15525(1)<br>Q9NR55(1)	uncertain
CPX-5342	RXRalpha-NCOA1 activated retinoic acid receptor complex	2	P19793(2)<br>Q15788(2)	uncertain

Table S2. Classification of transcription factors in *H. sapiens*. The table provides a list of 74 TFs in *H. sapiens* that belong to the basic helix-loop-helix (bHLH) or basic leucine zipper (bZIP) classes. The classes of the TFs are determined using the JASPAR database. The column 'UniProt ID' provides the UniProt IDs for each of the 74 TFs, whereas 'TF name' gives the name of the TF.

UniProt ID	TF name	Class
Q9HBZ2	ARNT2	Basic helix-loop-helix factors (bHLH)
P50553	ASCL1	Basic helix-loop-helix factors (bHLH)
Q92858	ATOH1	Basic helix-loop-helix factors (bHLH)
Q8N100	ATOH7	Basic helix-loop-helix factors (bHLH)
O15516	CLOCK	Basic helix-loop-helix factors (bHLH)
Q6QHK4	FIGLA	Basic helix-loop-helix factors (bHLH)
P61296	HAND2	Basic helix-loop-helix factors (bHLH)
Q14469	HES1	Basic helix-loop-helix factors (bHLH)
Q9Y543	HES2	Basic helix-loop-helix factors (bHLH)
Q5TA89	HES5	Basic helix-loop-helix factors (bHLH)
Q96HZ4	HES6	Basic helix-loop-helix factors (bHLH)
Q9BYE0	HES7	Basic helix-loop-helix factors (bHLH)
Q9Y5J3	HEY1	Basic helix-loop-helix factors (bHLH)
Q9UBP5	HEY2	Basic helix-loop-helix factors (bHLH)
Q16665	HIF1A	Basic helix-loop-helix factors (bHLH)
P61244	MAX	Basic helix-loop-helix factors (bHLH)
O75030	MITF	Basic helix-loop-helix factors (bHLH)
Q9UH92	MLX	Basic helix-loop-helix factors (bHLH)
Q99583	MNT	Basic helix-loop-helix factors (bHLH)
A6NI15	MSGN1	Basic helix-loop-helix factors (bHLH)

P50539	MXI1	Basic helix-loop-helix factors (bHLH)
P01106	MYC	Basic helix-loop-helix factors (bHLH)
P04198	MYCN	Basic helix-loop-helix factors (bHLH)
P13349	MYF5	Basic helix-loop-helix factors (bHLH)
P23409	MYF6	Basic helix-loop-helix factors (bHLH)
P15172	MYOD1	Basic helix-loop-helix factors (bHLH)
P15173	MYOG	Basic helix-loop-helix factors (bHLH)
Q8TAK6	OLIG1	Basic helix-loop-helix factors (bHLH)
Q13516	OLIG2	Basic helix-loop-helix factors (bHLH)
Q7RTU3	OLIG3	Basic helix-loop-helix factors (bHLH)
O43680	TCF21	Basic helix-loop-helix factors (bHLH)
Q9UL49	TCFL5	Basic helix-loop-helix factors (bHLH)
Q01664	TFAP4	Basic helix-loop-helix factors (bHLH)
P19532	TFE3	Basic helix-loop-helix factors (bHLH)
P19484	TFEB	Basic helix-loop-helix factors (bHLH)
O14948	TFEC	Basic helix-loop-helix factors (bHLH)
P22415	USF1	Basic helix-loop-helix factors (bHLH)
Q15853	USF2	Basic helix-loop-helix factors (bHLH)
P15336	ATF2	Basic leucine zipper factors (bZIP)
P18847	ATF3	Basic leucine zipper factors (bZIP)
P18848	ATF4	Basic leucine zipper factors (bZIP)
P17544	ATF7	Basic leucine zipper factors (bZIP)
O14867	BACH1	Basic leucine zipper factors (bZIP)
Q9BYV9	BACH2	Basic leucine zipper factors (bZIP)
Q16520	BATF	Basic leucine zipper factors (bZIP)
Q9NR55	BATF3	Basic leucine zipper factors (bZIP)
P49715	CEBPA	Basic leucine zipper factors (bZIP)
P17676	CEBPB	Basic leucine zipper factors (bZIP)
P49716	CEBPD	Basic leucine zipper factors (bZIP)
Q15744	CEBPE	Basic leucine zipper factors (bZIP)
P53567	CEBPG	Basic leucine zipper factors (bZIP)
P16220	CREB1	Basic leucine zipper factors (bZIP)
O43889	CREB3	Basic leucine zipper factors (bZIP)
Q03060	CREM	Basic leucine zipper factors (bZIP)
Q10586	DBP	Basic leucine zipper factors (bZIP)
P01100	FOS	Basic leucine zipper factors (bZIP)
P15407	FOSL1	Basic leucine zipper factors (bZIP)
P15408	FOSL2	Basic leucine zipper factors (bZIP)

Q16534	HLF	Basic leucine zipper factors (bZIP)
Q8WYK2	JDP2	Basic leucine zipper factors (bZIP)
P05412	JUN	Basic leucine zipper factors (bZIP)
P17275	JUNB	Basic leucine zipper factors (bZIP)
P17535	JUND	Basic leucine zipper factors (bZIP)
O75444	MAF	Basic leucine zipper factors (bZIP)
Q8NHW3	MAFA	Basic leucine zipper factors (bZIP)
Q9ULX9	MAFF	Basic leucine zipper factors (bZIP)
O15525	MAFG	Basic leucine zipper factors (bZIP)
O60675	MAFK	Basic leucine zipper factors (bZIP)
Q16621	NFE2	Basic leucine zipper factors (bZIP)
Q16649	NFIL3	Basic leucine zipper factors (bZIP)
P54845	NRL	Basic leucine zipper factors (bZIP)
Q10587	TEF	Basic leucine zipper factors (bZIP)
P17861	XBP1	Basic leucine zipper factors (bZIP)
Q16656	NRF1	Basic leucine zipper factors (bZIP)

Table S3. A list of 17 complexes from the set of 617 complexes in *S. cerevisiae* such that all the protein subunits of these complexes are transcription factors. 'Complex ID' is the identifier of the complex as given in the EBI Complex Portal database. The 617 complexes in *S. cerevisiae* were obtained from the EBI Complex Portal database. The list of TFs was obtained from the Yeastract database. 'Complex name' is the name of the complex as given in the EBI Complex Portal database. 'Size of the complex' is the number of protein subunits the complex is constituted of. 'Uniprot ID of protein subunits' gives the Uniprot IDs of the subunits in a complex along with their stoichiometric coefficients as integers within brackets. Stoichiometric coefficients which are unknown are represented by $(-)$. 'Is a TR' column is 'yes' if the complex acts as a transcriptional regulator (TR) based on manual literature curation. For a given complex, if all subunits show evidence for both TR binding and effects on expression, then its entry is 'yes' in the column 'TR binding and expression evidence', otherwise, the entry is 'no'. There are 11 complexes for which all protein subunits show evidence for both DNA binding and effects on expression.

Complex ID	Complex name	Size of the complex	Uniprot ID of protein subunits	Is a TR	TR binding and expression evidence
CPX-575	Ste12/Dig1/Dig2 transcription regulation complex	3	P13574(-) Q03063(-) Q03373(-)	uncertain	no
CPX-576	Tec1/Ste12/Dig1 transcription regulation complex	3	P13574(-) P18412(-) Q03063(-)	yes	no
CPX-828	RTG transcription factor complex	2	P32607(1) P38165(1)	yes	yes
CPX-946	SBF transcription complex	2	P09959(1) P25302(1)	yes	no
CPX-950	MBP transcription complex	2	P09959(-) P39678(-)	yes	no
CPX-999	MET4-MET28-MET31 sulfur metabolism transcription factor complex	3	P32389(-) P40573(-) Q03081(-)	yes	yes
CPX-1015	MET4-MET28-MET32 sulfur metabolism transcription factor complex	3	P32389(-) P40573(-) Q12041(-)	yes	yes
CPX-1016	CBF1-MET4-MET28 sulfur metabolism transcription factor complex	3	P17106(2) P32389(-) P40573(-)	yes	yes
CPX-1038	PIP2-OAF1 transcription factor complex	2	P39720(1) P52960(1)	yes	yes
CPX-1042	GAL3-GAL80 transcription regulation complex	2	P04387(2) P13045(2)	yes	no
CPX-1044	GAL4-GAL80 transcription repressor complex	2	P04386(2) P04387(2)	yes	yes
CPX-1200	RAP1-GCR1 transcription activation complex	2	P07261(2) P11938(-)	yes	yes
CPX-1229	RAP1-GCR1-GCR2 transcription activation complex	3	P07261(2) P11938(-) Q01722(2)	yes	yes
CPX-1277	INO2-INO4 transcription activation complex	2	P13902(-) P26798(-)	yes	yes
CPX-1415	IME1-UME6 transcription activation complex	2	P21190(-) P39001(-)	yes	yes
CPX-1663	CYP8-TUP1 corepressor complex	2	P14922(1) P16649(4)	corepressor	no
CPX-1830	CCAAT-binding factor complex	4	P06774(1) P13434(1) P14064(1) Q02516(1)	yes	yes

Table S4. Classification of transcription factors in *S. cerevisiae* The table provides a list of 17 TFs from the Yeastract database, that belong to the basic helix-loop-helix (bHLH) or basic leucine zipper (bZIP) classes. The classes of the TFs are determined using the JASPAR database. The column 'UniProt ID' provides the UniProt IDs for each of the 17 TFs, whereas 'TF name' corresponds to the name of the TF. All the 17 TFs shown in the table display evidence for DNA binding as well as effects on expression.

UniProt ID	TF Name	DNA binding and expression evidence	Class
P33122	TYE7	Yes	Basic helix-loop-helix factors (bHLH)
P38165	RTG3	Yes	Basic helix-loop-helix factors (bHLH)
P13902	INO4	Yes	Basic helix-loop-helix factors (bHLH)
P26798	INO2	Yes	Basic helix-loop-helix factors (bHLH)
P07270	PHO4	Yes	Basic helix-loop-helix factors (bHLH)
P17106	CBF1	Yes	Basic helix-loop-helix factors (bHLH)
P14164	ABF1	Yes	Basic helix-loop-helix factors (bHLH)
P32389	MET4	Yes	Basic leucine zipper factors (bZIP)
P40573	MET28	Yes	Basic leucine zipper factors (bZIP)
P41546	HAC1	Yes	Basic leucine zipper factors (bZIP)
P03069	GCN4	Yes	Basic leucine zipper factors (bZIP)
Q02100	SKO1	Yes	Basic leucine zipper factors (bZIP)
Q06596	ARR1	Yes	Basic leucine zipper factors (bZIP)
Q08182	YAP7	Yes	Basic leucine zipper factors (bZIP)
Q03935	YAP6	Yes	Basic leucine zipper factors (bZIP)
P40574	YAP5	Yes	Basic leucine zipper factors (bZIP)
P38749	YAP3	Yes	Basic leucine zipper factors (bZIP)

Table S5. Comparison of the fractions of four types of biologically meaningful BFs and the fraction of the BFs allowed by the most restrictive composition structures for $k \leq 5$ inputs. The fractions are computed with respect to all possible BFs for $k \leq 5$ inputs. The four types of biologically meaningful BFs include Unate functions (UFs), Canalyzing functions (CFs), Nested canalyzing functions (NCFs) and Read-once functions (RoFs). The column 'Composed BF' represents BFs contained in the most restrictive composition structure for a given k. The most restrictive composition structure is the composition structure that has the least number of BFs when compared to other composition structures with the same number of inputs k. For $k = 1$ and 2, there are no restrictions in possible BFs due to composition structure since only trivial composition structures such as $\{1\}$, $\{1,1\}$ and $\{2\}$ exist. For $k = 3$, 4 and 5, the most restrictive composition structures are $\{1,2\}$, $\{2,2\}$ and $\{2,3\}$, respectively.

Inputs (k)	Fraction of				
	UF	CF	NCF	RoF	Composed BF
1	1	1	0.5	0.5	1
2	0.875	0.875	0.5	0.5	1
3	0.406	0.469	0.25	0.25	0.594
4	0.033	0.054	0.011	0.013	0.018
5	5.37×10^{-5}	3.01×10^{-4}	2.47×10^{-6}	3.52×10^{-6}	1.67×10^{-5}

Table S6. Fraction of BFs in different composition structures that display biologically meaningful properties. The fraction of BFs in different non-trivial composition structures that also belong to each of the four types of biologically meaningful BFs, namely Unate functions (UFs), Canalyzing functions (CFs), Nested canalyzing functions (NCFs) and Read-once functions (RoFs). The fraction is computed with respect to all BFs allowed by a composition structure.

Composition structure	Fraction of biologically meaningful BFs in composition structure			
	UF	CF	NCF	RoF
{1,2}	0.632	0.789	0.421	0.421
{1,3}	0.249	0.722	0.151	0.151
{2,2}	0.525	0.604	0.185	0.265
{1,1,2}	0.220	0.298	0.118	0.134
{1,4}	0.022	0.672	0.006	0.007
{2,3}	0.191	0.469	0.046	0.095
{1,1,3}	0.099	0.307	0.040	0.054
{1,2,2}	0.177	0.250	0.055	0.099
{1,1,1,2}	0.018	0.036	0.003	0.004

Table S7. Number and fraction of BFs with odd bias in different composition structures. The fractions of BFs with odd bias in a composition structure are computed with respect to all allowed BFs in the composition structure. The column ‘Number of composed BFs’ gives the number of allowed BFs in a composition structure. The column ‘Odd biases present’ gives the list of odd biases of BFs that are present in a composition structure. Note that the table only gives data for non-trivial composition structures with $k \leq 5$ inputs.

Composition structure	Number of composed BFs	BFs with odd bias in composition structure		
		Number	Fraction	Odd biases present
{1,2}	152	64	0.421	1,3
{1,3}	4864	1760	0.361	1,3,5,7
{2,2}	1208	320	0.264	1,3,7
{1,1,2}	6216	2368	0.381	1,3,5,7
{1,4}	1921928	646144	0.336	1,3,5,7,9,11,13,15
{2,3}	71608	17024	0.238	1,3,5,7,9,11,15
{1,1,3}	263488	75584	0.287	1,3,5,7,9,11,13,15
{1,2,2}	100768	25344	0.252	1,3,5,7,9,11,13,15
{1,1,1,2}	3446488	1266944	0.368	1,3,5,7,9,11,13,15

Table S8. Enrichment of composed BFs in the reference biological dataset. The enrichment factors for composed BFs in different non-trivial composition structures with number of inputs $k \leq 5$ and the associated one-sided p-values.

**Composition structure**	**Enrichment factor**	p**-value**
{1,2}	1.63	6.15×10^{-72}
{1,3}	12.48	4.33×10^{-245}
{2,2}	40.37	3.90×10^{-274}
{1,1,2}	10.30	7.57×10^{-250}
{1,4}	1948.23	0
{2,3}	45760.08	0
{1,1,3}	14732.62	0
{1,2,2}	36887.66	0
{1,1,1,2}	1158.31	0

Table S9. p**-values corresponding to the relative enrichment values of biologically meaningful BFs within different composition structures.** The p-values corresponding to the relative enrichment values E_R for the four biologically meaningful sub-types of BFs within different composition structures with number of inputs $k \leq 5$. The four biologically meaningful sub-types within composed BFs include those BFs in a composition structure that also happen to be Unate functions (UFs), Canalyzing functions (CFs), Nested canalyzing functions (NCFs), or Read-once functions (RoFs).

**Composition structure**	p**-values corresponding to** E_R **of sub-types in composition structure**			
	**UF**	**CF**	**NCF**	**RoF**
{1,2}	0	0	1.79×10^{-115}	1.79×10^{-115}
{1,3}	0	0	2.27×10^{-176}	2.27×10^{-176}
{2,2}	1.75×10^{-54}	1.98×10^{-24}	5.61×10^{-100}	3.74×10^{-96}
{1,1,2}	3.08×10^{-166}	1.30×10^{-105}	1.42×10^{-185}	4.53×10^{-202}
{1,4}	5.19×10^{-227}	0	2.26×10^{-254}	3.13×10^{-258}
{2,3}	0	1.74×10^{-27}	1.62×10^{-111}	6.60×10^{-105}
{1,1,3}	0	1.26×10^{-57}	7.92×10^{-146}	8.03×10^{-160}
{1,2,2}	0	4.12×10^{-64}	7.77×10^{-123}	2.05×10^{-118}
{1,1,1,2}	0	1.32×10^{-181}	2.18×10^{-277}	9.30×10^{-301}

Table S10. **p-values for comparison between the enrichments of composed BFs and biologically meaningful BFs with minimum complexity in the reference biological dataset.** T_C denotes the set of composed BFs allowed by a composition structure at a given number of inputs k, T_{NCF} denotes the set of all k-input nested canalyzing functions (NCFs), and T_{RoF} denotes the set of all k-input Read-once functions (RoFs). $\cap$ represents the intersection of two sets and $\setminus$ represents the set-theoretic difference. "–" in the columns $T_{NCF} \setminus T_C$ or $T_{RoF} \setminus T_C$ indicates that the NCFs or RoFs are a subset of the set of BFs allowed by the composition structure.

**Composition structure**	$T_C \cap T_{NCF}$	$T_C \setminus T_{NCF}$	$T_{NCF} \setminus T_C$	$T_C \cap T_{RoF}$	$T_C \setminus T_{RoF}$	$T_{RoF} \setminus T_C$
{1,2}	3.09×10^{-184}	1	–	3.09×10^{-184}	1	–
{1,3}	0	0.966	–	0	0.966	1.66×10^{-19}
{2,2}	0	1.59×10^{-11}	7.07×10^{-70}	0	0.009	7.07×10^{-70}
{1,1,2}	0	0.406	–	0	0.999	–
{1,4}	0	2.17×10^{-35}	–	0	7.77×10^{-26}	1.06×10^{-47}
{2,3}	0	9.10×10^{-80}	4.44×10^{-106}	0	4.83×10^{-30}	8.09×10^{-105}
{1,1,3}	0	2.87×10^{-67}	–	0	8.85×10^{-25}	3.43×10^{-05}
{1,2,2}	0	1.36×10^{-76}	1.31×10^{-30}	0	1.00×10^{-28}	1.31×10^{-30}
{1,1,1,2}	0	7.82×10^{-52}	–	0	1.51×10^{-22}	–

Table S11. **Comparison between the enrichments of composed BFs and biologically meaningful BFs in the reference biological dataset.** The table provides the enrichment factors of composed BFs allowed by non-trivial composition structures with $k \leq 5$ inputs and of two biologically meaningful BFs namely unate functions (UFs) and canalyzing functions (CFs). T_C denotes the set of composed BFs allowed by a composition structure at a given number of inputs k, whereas T_{UF} and T_{CF} denote the set of all k-input UFs and CFs, respectively. $\cap$ represents the intersection of two sets and $\setminus$ represents the set-theoretic difference. "–" in the column $T_{CF} \setminus T_C$ indicates that the CFs are a subset of the set of BFs allowed by the corresponding composition structure.

**Composition structure**	$T_C \cap T_{UF}$	$T_C \setminus T_{UF}$	$T_{UF} \setminus T_C$	$T_C \cap T_{CF}$	$T_C \setminus T_{CF}$	$T_{CF} \setminus T_C$
{1,2}	2.58	0.00	1.01	2.06	0.00	–
{1,3}	50.17	0.00	4.76	17.28	0.00	–
{2,2}	76.53	0.44	10.91	61.59	7.97	5.66
{1,1,2}	46.54	0.05	1.91	32.54	0.87	0.31
{1,4}	89183.19	14.64	2623.97	2897.47	0.00	–
{2,3}	239564.87	0.00	4316.45	90144.74	6518.64	568.71
{1,1,3}	148416.79	0.00	1616.48	44868.92	1357.79	90.92
{1,2,2}	208387.45	0.00	2329.87	136371.86	3645.06	239.02
{1,1,1,2}	64895.59	0.00	1303.10	30112.53	91.11	47.07

Table S12. **p-values for comparison between the enrichments of composed BFs and biologically meaningful BFs in the reference biological dataset.** The table provides the enrichment factors of composed BFs allowed by non-trivial composition structures with $k \leq 5$ inputs and of two biologically meaningful BFs namely, unate functions (UFs) and canalyzing functions (CFs). T_C denotes the set of composed BFs allowed by a composition structure at a given number of inputs k, whereas T_{UF} and T_{CF} denote the set of all k-input UFs and CFs, respectively. $\cap$ represents the intersection of two sets and $\setminus$ represents the set-theoretic difference. "–" in the column $T_{CF} \setminus T_C$ indicates that the CFs are a subset of the set of BFs allowed by the corresponding composition structure.

**Composition structure**	$T_C \cap T_{UF}$	$T_C \setminus T_{UF}$	$T_{UF} \setminus T_C$	$T_C \cap T_{CF}$	$T_C \setminus T_{CF}$	$T_{CF} \setminus T_C$
{1,2}	3.23×10^{-149}	1	0.413	1.83×10^{-111}	1	–
{1,3}	0	0.999	1.39×10^{-08}	8.86×10^{-279}	0.995	–
{2,2}	0	0.661	5.93×10^{-49}	1.14×10^{-280}	1.39×10^{-10}	1.02×10^{-29}
{1,1,2}	0	0.999	0.041	0	0.652	0.960
{1,4}	0	0.002	2.02×10^{-59}	0	0.023	–
{2,3}	0	0.002	3.64×10^{-116}	0	5.16×10^{-36}	5.39×10^{-66}
{1,1,3}	0	0.009	1.99×10^{-38}	0	3.34×10^{-29}	1.24×10^{-09}
{1,2,2}	0	0.003	1.93×10^{-58}	0	1.16×10^{-36}	1.17×10^{-25}
{1,1,1,2}	0	0.116	2.68×10^{-26}	0	1.17×10^{-20}	1.22×10^{-05}
